# An inverse association between plasma benzoxazinoid metabolites and PSA after rye intake in men with prostate cancer revealed with a new method

**DOI:** 10.1038/s41598-022-08856-z

**Published:** 2022-03-28

**Authors:** Elise Nordin, Stine K. Steffensen, Bente B. Laursen, Sven-Olof Andersson, Jan-Erik Johansson, Per Åman, Göran Hallmans, Michael Borre, Dan Stærk, Kati Hanhineva, Inge S. Fomsgaard, Rikard Landberg

**Affiliations:** 1 Division of Food and Nutrition Science, Department of Biology and Biological Engineering, Chalmers University of Technology, 412 39 Gothenburg, Sweden; 2 Department of Agroecology, Aarhus University, Forsøgsvej 1, 4200 Slagelse, Denmark; 3 Department of Urology, Faculty of Medicine and Health, Örebro University, Örebro, Sweden; 4 Department of Molecular Sciences, Swedish University of Agricultural Sciences, Box 7015, Uppsala, Sweden; 5 Department of Public Health and Clinical Medicine, Umeå University, Umeå, Sweden; 6 Department of Urology, Aarhus University Hospital, Palle Juul-Jensens Boulevard 99, 8200 Aarhus N, Denmark; 7 Department of Drug Design and Pharmacology, Faculty of Health and Medical Sciences, University of Copenhagen, Universitetsparken 2, 2100 Copenhagen, Denmark; 8 Department of Life Technologies, Food Chemistry and Food Development Unit, 20520 Turku, Finland; 9 School of Medicine, Institute of Public Health and Clinical Nutrition, University of Eastern Finland, 70210 Kuopio, Finland

**Keywords:** Cancer, Chemical biology, Molecular medicine, Risk factors, Chemistry

## Abstract

Prostate cancer (PC) is a common cancer among men, and preventive strategies are warranted. Benzoxazinoids (BXs) in rye have shown potential against PC in vitro but human studies are lacking. The aim was to establish a quantitative method for analysis of BXs and investigate their plasma levels after a whole grain/bran rye vs refined wheat intervention, as well as exploring their association with PSA, in men with PC. A quantitative method for analysis of 22 BXs, including novel metabolites identified by mass spectrometry and NMR, was established, and applied to plasma samples from a randomized crossover study where patients with indolent PC (n = 17) consumed 485 g whole grain rye/rye bran or fiber supplemented refined wheat daily for 6 wk. Most BXs were significantly higher in plasma after rye (0.3–19.4 nmol/L in plasma) vs. refined wheat (0.05–2.9 nmol/L) intake. HBOA-glc, 2-HHPAA, HBOA-glcA, 2-HPAA-glcA were inversely correlated to PSA in plasma (*p* < 0.04). To conclude, BXs in plasma, including metabolites not previously analyzed, were quantified. BX metabolites were significantly higher after rye vs refined wheat consumption. Four BX-related metabolites were inversely associated with PSA, which merits further investigation.

## Introduction

Prostate cancer is the second most common cancer in men worldwide with high mortality in many Western countries in Europe and in the US^1^. In particular, the disease has high prevalence in the Nordic countries, partly due to increased incidence during -90ies, as a likely result of introduction of prostate specific antigen (PSA) screening^1^. The high prevalence calls for research efforts to develop preventive strategies adaptable to the Nordic region. The causes of prostate cancer are yet to be explained and there are only a few well-established risk factors, such as age, race, family history of prostate cancer and some genetic variants^2^. Lifestyle, including diet, has been suggested as potential modifiable risk factor and there is strong evidence that overweight, or obesity increase the risk of advanced prostate cancer^3^. However, for specific dietary factors the evidence is limited and controversial^4^. Rye-based foods or foods rich in components from rye are of high relevance in the Nordic region and have attracted interest for prevention and mitigation of prostate cancer^5,6^. Rye is rich in bioactive compounds and is the cereal with highest dietary fibre content^6^. Rye-based foods have been suggested to reduce risk of developing prostate cancer in epidemiological studies as well as to reduce initial tumor progression and reduce prostate-specific antigen (PSA) in animal model studies and in a few small human studies^7–14^. The potential mechanism for the preventive effect has been suggested to be related to lowered insulin secretion caused by rye^14^, high intake of lignans^15^, and more recently, due to benzoxazinoids (BXs) and their metabolites. BXs represent a group of compounds with high bioactive potential present in rye and to some extent in wheat and maize^12,16–19^. In planta, BXs are frequently found as conjugated forms, most typically with glucose and are produced for defense and allelopathic purposes^20^. The most common chemical structures of BXs include hydroxamic acids (2,4-dihydroxy-1,4-benzoxazin-3-one, DIBOA; 2,4-dihydroxy-7-methoxy-1,4-benzoxazin-3-one, DIMBOA), lactams (2-hydroxy-1,4-benzoxazin-3-one, HBOA; 2-hydroxy-7-methoxy-1,4-benzoxazin-3-one, HMBOA) and benzoxazolinones (1,3-benzoxazolin-2-one, BOA; 6-methoxy-1,3-benzoxazolin-2-one, MBOA). Hydroxamic acids and lactams typically occur as sugar- or double sugar conjugates.

BXs have been reported in various in vitro studies to possess antimicrobial, anticancer, reproductive system stimulatory, central nervous system stimulatory, and immunoregulatory activities^19^. BXs have been hypothesized to affect growth of prostate cancer cells, which could be one mechanism partly explaining the observed effects of rye on biomarkers of prostate cancer progression^12^. Several in vitro studies have found an inhibitory effect on prostate cancer cells for the BXs DIBOA and HMBOA^21–24^. Although a few small dietary intervention studies have shown promising suggestive results including lower PSA and slower initial tumor growth after rye vs control^14,25^, no study has been reported where the effects have been linked to BX intake or BX metabolite concentrations in biological fluids. In a small pilot study with 10 prostate cancer patients, it was shown that rye-enriched diet rich in BXs for 1 week prior to prostatectomy resulted in detectable BXs concentrations in prostate tissue (0.15–10.6 ng/g tissue)^12^. Occurrence of BXs and BX-derived metabolites in human and animal plasma has been reported before^12,26–31^ but the isolation of mammal BX-derived metabolites from biofluids and structural elucidation by NMR has never been reported before and thus quantification of BX-metabolites in plasma have therefore not yet been reported. Establishment of a quantitative method of BX and their metabolites in human samples is an important step for further investigations of the role of BX in human health.

The aim of our current study was to establish a quantitative method for comprehensive analysis of BX and their common metabolites in plasma samples from humans. The aim was further to apply the method to evaluate the effect of a diet high in whole grain/bran rye-based foods rich in BXs vs fiber supplemented refined wheat on the structural and quantitative variety of BXs and BX-derived metabolites in plasma samples after regular consumption, and to investigate whether the concentrations of any of such metabolites correlated with PSA levels in men with indolent prostate cancer.

## Methods

### Chemicals

Chemicals used for sample preparation included: Methanol (Fisher Scientific), Milli-Q water (from a Milli-Q Advantage A10 instrument with LC-pack (Merck Millipore, Darmstadt, Germany), formic acid, hydrochloric acid, ammonium hydroxide, trifluoro acetic acid, acetic acid (all chemicals from Merck).

HPLC-grade solvents, methanol (Rathburn, Walkerburn, Scotland), acetonitrile (Fisher Scientific, Denmark), 2-propanol (Fisher Scientific) and Milli-Q water (Merck Millipore, Darmstadt, Germany) were used in the LC-MSMS analysis.

Reference standards used for the analysis are listed in Table 1. The standards were obtained as follows: Group 1 (compounds known to be present in rye): BOA and MBOA were purchased from Sigma-Aldrich. HBOA-glc, DIBOA, HMBOA-glc, HBOA, HMBOA, HBOA-glc-hex, DIBOA-glc, and DIBOA-glc-hex were obtained as part of an on-going patenting process. DIMBOA-glc was obtained as described by Pedersen et al.^32^. Group 2 (compounds known as phase 1 metabolites of BXs): 2-HPAA, HPMA, 2-HHPAA, and HMPAA were purchased from AKOS Consulting and Solutions, Germany. Group 3 (phase 2 metabolites of BXs): HBOA-glcA, 2-HPAA-glcA, 2-HHPAA-glcA, 2-HPAA-sulfate, 2-HHPAA-sulfate, and DIBOA-sulfate were isolated from urine as described below. Group 4 (non-BX related commercially available standards used for semi-quantification of Group 3 compounds): 4-HPAA-sulfate (known as acetaminophen-sulfate) and 4-HPAA-glcA (known as acetaminophen-glucuronic acid) were purchased from Sigma-Aldrich. The internal standard d_3_-MBOA was obtained as described by Etzerodt et al.^33^.

**Table 1:** Acronym, systematic name, and structure of compounds quantified in plasma samples together with MRM transitions (upper value for each compound denotes the quantifier transition, lower value denotes qualifier transition), and compound dependent parameters used in the LC–MS/MS analysis.

Group no^a^	Acronym	Systematic name	Structure	Q1 (m/z)	Q3 (m/z)	DP (V)	CE (V)	CXP (V)	Retention time^1^	Window (s)
1 C	HBOA-glc	2-β-D-glucopyranosyloxy-1,4-benzoxazin-3-one		325.9	163.9	− 85	− 22	− 13	5.3	25
				325.9	107.9	− 85	− 46	− 13		
1 C	DIBOA	2,4-dihydroxy-1,4-benzoxazin-3-one		180	133.97	− 40	− 10	− 13	6.3	25
				134	41.933	− 70	− 48	− 13		
1 C	DIMBOA	2,4-dihydroxy-7-methoxy-1,4-benzoxazin-3-one		164	148.9	− 60	− 20	− 13	7.0	120
				164	121	− 60	− 28	− 13		
1 C	HMBOA-glc	2-β-D-glucopyranosyloxy-7-methoxy-1,4-benzoxazin-3-one		355.97	193.9	− 95	− 22	− 13	6.0	25
				355.97	138	− 95	− 38	− 13		
1 A	BOA	benzoxazolin-2-one		133.9	41.93	− 70	− 48	− 13	7.7	25
				133.9	91.13	− 70	− 26	− 13		
1 A	MBOA	6-methoxybenzoxazolin-2-one		163.9	148.9	− 45	− 20	− 13	8.2	25
				163.9	121	− 45	− 28	− 13		
1 A	HBOA	2-hydroxy-1,4-benzoxazin-3-one		163.91	107.93	− 60	− 22	− 13	6.4	25
				163.91	107	− 60	− 40	− 13		
1 A	HMBOA	2-hydroxy-7-methoxy-1,4-benzoxazin-3-one		193.9	122.9	− 65	− 28	− 13	7.0	25
				193.9	138	− 65	− 18	− 13		
1 L	HBOA-glc-hex^b^	2-[4-*O*-hexosepyranosyl-β-D-glucopyranosyl]oxy-1,4-benzoxazin-3-one		488	164	− 105	− 30	− 13	4.5	25
				488	108	− 105	− 65	− 13		
1 L	DIBOA-glc	2-β-D-glucopyranosyloxy-4-hydroxy-1,4-benzoxazin-3-one		341.9	133.8	− 75	− 24	− 13	5.2	25
				341.9	180	− 75	− 14	− 13		
1 L	DIBOA-glc-hex^b^	2-[4-*O*-hexosepyranosyl-β-D-glucopyranosyl]oxy-4-hydroxy-1,4-benzoxazin-3-one		504	133.9	− 85	− 52	− 13	4.5	25
				504	162	− 85	− 52	− 13		
1 L	DIMBOA-glc	2-β-D-glucopyranosyloxy-4-hydroxy-7-methoxy-1,4-benzoxazin-3-one		372	148.8	− 80	− 40	− 13	6.2	25
				372	164	− 80	22	− 13		
2 HH	2-HPAA	*N*-(2-hydroxyphenyl)-acetamide		149.9	107.9	− 55	− 22	− 123	6.0	25
				149.9	107	− 55	− 38	− 13		
2 HH	HPMA	*N*-(2-hydroxyphenyl)-malonamic acid		193.9	149	− 30	− 10	− 13	5.4	
				150	107.9	− 55	− 22	− 13		25
2 HH	2-HHPAA	2-hydroxy-*N*-(2-hydroxyphenyl)-acetamide		166	108	− 62	− 26	− 13	5.5	
				166	118	− 62	− 18	− 13		25
2 HH	2-HMPAA	*N*-(2-hydroxy-4-methoxyphenyl)-acetamide		180	138	− 70	− 10	− 13	6.7	25
				180	122	− 70	− 38	− 13		
3	HBOA-glcA	2-glucuronopyranosyloxy-1,4-benzoxazin-3-one		340	134	− 70	− 20	− 13	5.0	45
				340	108	− 50	− 60	− 13		
3	2-HPAA-glcA	*N*-(2-glucuronopyranosyloxy-phenyl)-acetamide		326	150	− 60	− 25	− 13	4.5	25
				326	108	− 65	− 48	− 13		
3	2-HHPAA-glcA	2-hydroxy*-N*-(2-β-D-glucuronopyranosyloxy-phenyl)-acetamide		342	166	− 70	− 30	− 13	3.9	25
				342	118	− 50	− 30	− 13		
3	2-HPAA-sulfate	2-acetamidophenyl sulfate		230	150	− 65	− 38	− 13	4.0	25
				230	108	− 65	− 38	− 13		
3	2-HHPAA-sulfate	2-(2′-hydroxyacetamido)-phenyl sulfate		246	166	− 50	− 20	− 13	3.4	25
				246	118	− 50	− 38	− 13		
3	DIBOA-sulfate	4-hydroxy-1,4-benzoxazin-3-one-2-yl sulfate		260	108	− 65	− 38	− 13	4.3	25
				260	180	− 20	− 20	− 13		
4	4-HPAA-sulfate	4-acetamidophenyl sulfate		229.9	107.1	− 60	− 48	− 7	4.1	45
				229.9	150	− 65	− 38	− 13		
4	4-HPAA-glcA	*N*-(4-β-D-glucuronopyranosyloxy-phenyl)-acetamide		325.9	150	− 60	− 25	− 13	2.4	25
				325.9	107	− 65	− 48	− 13		

Qualifier MRM transitions were not determined for Group 2 compounds (lower 6 compounds).

^a^Compounds from group 1 and 2: LC–MS/MS MRM method 1; Compounds from group 3 and 4: LC–MS/MS MRM method 2. Letters A, C, L, HH show which BX compounds were mixed in each of the four standard mixtures.

^b^The structure of the second hexose has not yet been fully elucidated although the structure is depicted with Glc as the second hexose moiety.

### Isolation and purification of BX metabolites from a urine lot high in BX for structural confirmation

For structural determination of novel BXs using data dependent acquisition-mass spectrometry (DDA-MS) and nuclear magnetic resonance (NMR), samples of high concentration are needed. Therefore, urine samples from an already performed clinical trial with rye was used. Ten volunteers had a diet rich in rye, comprising six slices of rye bread a day or equivalent for one week. Before and after the rye intervention, 24 h urine samples were collected from the 10 participants^12^. The post- intervention urine samples were combined into one lot and stored at − 20 °C until initiation of the purification work. The combined urine was passed through MF-Millipore Membrane Filters (0.45 µm, Merck) to remove precipitated matter. The precipitate was discarded after verifying with HPLC–MS that it did not contain significant amounts of BX metabolites.

Portions of 100 ml urine were diluted 1:1 with water prior to loading onto a conditioned and equilibrated 6 g, 35 cc HLB SPE column (Oasis HLB from Waters). The sample was applied through the column at approx. 1 drop per sec. to allow maximum interaction of the sample with the column material. At the end of the process, vacuum was applied to the column until no more drops appeared. The cartridge was eluted 2*20 ml water, then with 3*20 ml 5% methanol (aq) followed by 3*20 ml 20% methanol (aq) at 1 drop per s. The cartridge was emptied by vacuum between each fraction. During the loading procedure four consecutive fractions of 50 ml were collected; subsequent portions were collected individually and analyzed for the content of BX related metabolites using the HPLC–MS DDA-EMS-EPI and DDA- multiple reaction monitoring (MRM)-EPI^34^ (Supplementary Text 1 online).

The fractions from the HLB-SPE containing the BX compounds were dried and reconstituted in 20 ml 2.5% formic acid in water and loaded onto a cationic exchange SPE column (MCX Oasis, Waters, 6 cc, 500 mg) put in series before a weak anion exchange column (WAX Oasis, Waters, 6 cc, 500 mg). They were conditioned and equilibrated according to vendor recommendations prior to loading of the reconstituted sample. Further details can be found in Supplementary Text 2 online. The wash and elution sequence was as follows with a flow of 1 drop pr sec. and emptying of the cartridge by vacuum until no further drops appeared between each fraction: (a) 2*20 ml 2% formic acid (aq); (b) 3*20 ml 40% methanol (aq); (c) 4*20 ml 2% hydrochloric acid and 20% methanol in water; (d) 2*20 ml 2% hydrochloric acid and 40% methanol in water; (e) 2*20 ml 2% formic acid in methanol; (f) only through WAX, 3*20 ml 2% ammonium hydroxide and 40% methanol in water. Hydrochloric acid in step (c) and (d) was chosen for its acidity and volatility in combination with the chloride ion’s low affinity for the anion exchange material.

Semi-preparative HPLC was applied to the dried ad reconstituted SPE fractions. An Agilent HPLC equipped with a PDA detector (1100), a fraction collector (1260 infinity), pump (1260 infinity) was used for the isolation. Four UV-tracks (λ = 240, 254, 260, and 280 nm) were used for generating and evaluating the chromatogram and the fraction collector was set to collect by time in 30 s time windows. The separations were performed using a Synergy Fusion column (4 µm, 150*10 mm, Phenomenex). The eluents used were A: 0.5% trifluoracetic acid (aq) B: 95% methanol (aq). The chromatographic method was adjusted individually for the fractions; a typical example is the following: 0–9 min 12% B, at 21 min 35% B, at 25–28 min 100% B, 28.5–35 12% B. Collected fractions were analyzed for BX-related metabolites using the DDA-MS methods described in Supplementary Text 1 online. The fractions were combined accordingly. A second HPLC separation using the same system but a new column (Kinetex Biphenyl, 5 µm, 150*10 mm, Phenomenex) was needed to produce sufficiently pure compounds for NMR analysis. All fractions were dried in vacuum as soon as possible to avoid hydrolysis by the strong acid during storage. Fractions were analyzed by DDA-MS and NMR (Supplementary Text 1 and 3 online) and comparisons to customized synthesized standards were performed.

### Quantitative LC–MS/MS MRM analysis of BX and BX metabolites in plasma samples

After fraction collection of the target BX-metabolites from the urine samples, a method for their quantitative analysis in plasma was established. Plasma samples (see below) were taken from the − 80 °C freezer and thawed at 4 °C. After thorough mixing of each individual sample, 200 µl were taken from each sample and transferred to an Eppendorf tube and randomized into 6 batches and frozen at − 80 °C. One batch (approx. 40 samples) at a time (day) was thawed at 4 °C. All solvents and samples were kept in ice-bath throughout the whole procedure. Methanol (400 µl) with 4 ng/ml d_3_-MBOA was added to each Eppendorf and the mixture was thoroughly shaken. The samples were placed at − 20 °C for 30 min for optimization of the protein precipitation, followed by centrifugation at 10.000 g for 30 min. The supernatant was carefully removed and stored in glass vials at − 20 °C until analysis, when the samples were thawed, diluted 1:3 with Milli-Q water, filtered and placed in the autosampler in a dedicated vial.

The LC–MS system setup was similar as for qualitative analysis in urine (Supplementary Text 1 online) with the exception that the LC flow and gradient time points were adjusted to 500 µl/min and a total duration of 13.8 min for increased throughput. The suitability of the flow-dependent MS parameters was investigated but no adjustments were required. Compound dependent mass spectrometry parameters (Table 1) for MRM analysis in ESI negative mode were determined by introducing individual standards (Table 1, compounds from group 1, 2, and 4) to the electrospray source by direct infusion. The fragments associated with the deprotonated precursor molecule were determined and two sets of MRM transitions were selected, a quantifier and a qualifier (Table 1). The identity of analytes was confirmed by the comparison of quantifier/qualifier ratios and retention time of the compounds in the sample with those obtained from the standards.

Samples were analyzed in duplicates whit a few exceptions where sample amount did not allow for it. The quantification of the standards in group 1 and 2 shown in Table 1 was performed on basis of the quantifiers in relation to standard curves (mixtures A, C, L, HH, Table 1) with concentrations ranging from 0.006 ng/ml to 50 ng/ml with quadratic regression and 1/x weighing. The internal standard d_3_-MBOA was used for continuous control of the sensitivity of the instrument. The samples were distributed randomly and analyzed in 6 batches.

Semi-quantification of the Phase 2 metabolites in group 3 (Table 1) was performed on basis of the quantifiers in relation to the standard curves of standards with similar structural features (Table 1). Out of the potential Phase 2 BX metabolites HBOA-glcA was semi-quantified against HBOA-glc; 2-HPAA-glcA and 2-HHPAA-glcA were semi-quantified against 4-HPAA-glcA; 2-HPAA-sulfate, 2-HHPAA-sulfate and DIBOA-sulfate were semi-quantified against 4-HPAA-sulphate. The standard curves were prepared in the concentration range 0.006–100 ng/ml in 20% methanol and 0.25% acetic acid in water and used 1/x weighted and with quadratic regression. Method validation is described in Supplementary Text 4 online.

### Study design, participants, diet, and samples

The method for quantitative analysis of BXs and their metabolites in plasma was applied on samples from a randomized crossover study with two 6-wk intervention periods separated by a 2-wk washout period in men with indolent prostate cancer. Participants and study design have been described in detail elsewhere^14^. In brief, 17 men with biopsy-proven, low-grade, untreated prostate cancer were included in the study. All participants were in the age of 73.5 ± 4.6 years (mean ± standard deviation) with a BMI of 27.5 ± 4.6 kg/m^2^. The study was carried out with approval from the Regional Ethics Review Board in the Uppsala Region (DnR 2005/002). The study was conducted according to relevant guidelines, informed consent was obtained from all participants. During the study, participants were instructed to keep to their habitual diet except for bread, porridge, muesli, and other fiber- or lignan-rich foods and table spreads, which were replaced by products provided by the study (intervention foods). During the interventions, participants were provided isocaloric, dietary fiber adjusted intakes of whole grain/bran rye foods (RF) or refined wheat products with added cellulose derived from cereal straws (WP). Participants were asked to include intervention foods as part of their breakfast, lunch, dinner, and 2–3 snacks/day during the intervention weeks. In brief, the intervention foods consisted of 300 g/d soft bread (5 pieces), 100 g/d crisp bread (10 pieces), 50 g/d breakfast cereals, 35 g/d porridge (uncooked), and 58 g/d table spread. The bran that was added to the soft bread and crisp bread were sourdough fermented before addition. The exact nutritional composition of intervention food products has been described elsewhere^14^. The fiber intake from the experimental foods were very high, 58 g/day during the rye regime and 57 g/day during the endosperm fiber supplemented wheat regime^14^. BX contents of the intervention foods was not analyzed since there was no material left when the current study was initiated. For qualitative comparisons, we have estimated the contents based on previous human studies with similar products^28,35^. Blood samples were collected at baseline and after 2, 4, and 6 weeks in each period. Samples were processed immediately after collection as described elsewhere and were stored at − 80 °C until BX analysis^14^.

### Statistical analysis

The plasma levels of BX metabolites between treatments were evaluated by mixed linear model. Identity was included as a random factor and diet, week, and period as fixed factors. Initially, the interaction term diet x time was included but was not significant and therefore removed. There was no clear trend of a time effect, thus data was average for each id and treatment. Normality of the residual distribution of each variable was tested by visually inspecting the Q-Q plot and Residuals versus Fitted plot. Data where residuals were not normally distributed was log-transformed. If still not normally distributed data was rank transformed. Log-transformed values were back-transformed to the original scale. Overall effects were investigated with type III tests adjusted with the false discovery rate method (*p* < 0.05). Pairwise comparisons were performed for overall effects. Data is presented as mean and 95% confidence interval. Rank-transformed variables were presented as median and interquartile range.

Principal component analysis (PCA) was conducted on data averaged for each treatment and identity. Imputation of data for metabolites in the PCA was performed using LOD/2, the LOD was estimated by averaging the 3 lowest values for each variable divided by 2. The PCA was visualized with the R package rdCV (https://gitlab.com/CarlBrunius/rdCV, version 0.0.101). Partial spearman correlation was performed on variables significant between diets and included total PSA and metabolites adjusted for id, including all 8 data points (baseline, week 2, 4 and 6 for each diet). For each variable, the correlation with total PSA was based on complete data, excluding missing values. The analyses were performed in the programming software R (version 4.0.1), packages used were lme4 (1.1–27.1), car (3.0–11), emmeans (1.7.0) and Hmisc (4.6–0).

## Results and discussion

We identified several benzoxazinoids and their metabolites in urine from humans consuming high amounts of rye-based foods using data-dependent acquisition mass spectrometry DDA-MS where spectral data from known BX’s were compared to that of unknown peaks. Several novel BX metabolites were discovered and isolated from urine and then incorporated in a quantitative/semi-quantitative method for plasma samples. This method was then validated (Supplementary Text 4 online) and applied for analysis of plasma samples from a whole/grain rye intervention study in men with indolent prostate cancer.

### Novel BX-related metabolites in urine

**Figure 1:**
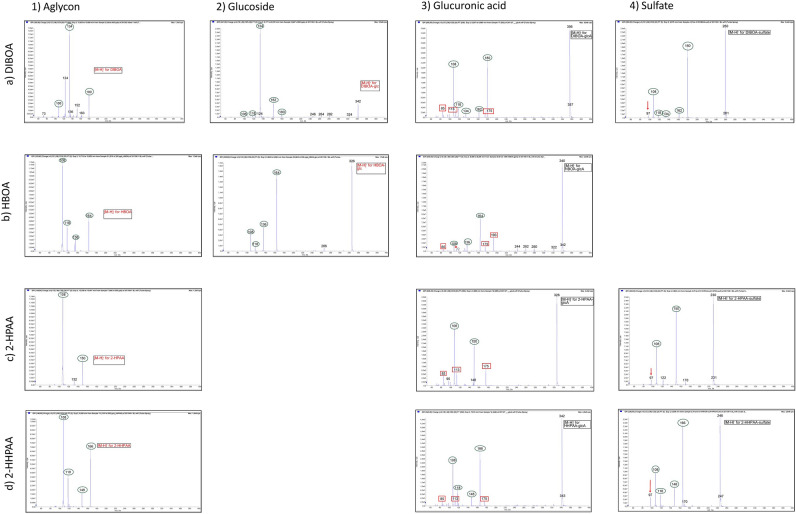
Mass spectra of standards (name in red) of DIBOA, DIBOA-glc, HBOA, HBOA-glc, 2-HPAA and 2-HHPAA; and the tentatively assigned phase 2 metabolites DIBOA-glcA, DIBOA-sulfat, HBOA-glcA*, 2-HPAA-glcA*, 2-HPAA-sulfat, 2-HHPAA-glcA*, and 2-HHPAA-sulfat. *Structure subsequently confirmed by NMR and comparison to custom synthesized standards.

The purification of BX-related metabolites from urine is presented in Supplementary Text 2 online. By utilizing urine samples that were high in BX-metabolite concentrations, we were able to identify the following BX compounds/metabolites: DIBOA-glcA, DIBOA-sulfate, HBOA-glcA, 2-HPAA-glcA, 2-HPAA-sulfate, 2-HHPAA-glcA, and 2-HHPAA-sulfate (For full names and structures see Table 1). The structures of DIBOA-glcA and DIBOA-sulfate were tentatively elucidated by comparison of their mass spectra to the mass spectra of the pure standards of DIBOA and of DIBOA-glc as seen in Fig. 1, a1 and a2 (zoomable in the electronic form, ‘Fig. S1’). Fragments (m/z 180/162/134/118/108) of the DIBOA moiety (marked with green spheres) can be universally recognized across the three spectra of DIBOA-conjugates and to some extent also for DIBOA itself, though DIBOA also displays fragments indicating radical fragmentation reactions (e.g. loss of 17 from a non-primary amine) probably due to its high reactivity. In the spectrum of DIBOA-glcA (Fig. 1, a3) common fragments^36^ of glucuronic acid (m/z 175/113/85, marked with red rectangles) can be seen, thus supporting the annotation. DIBOA-sulfate (Fig. 1, a4) likewise, shows a signal at m/z 97 corresponding to a HSO_4_^−^ ion and a neutral loss of 80 units corresponding to SO_3_^37^_._ Previously, DIBOA-sulfate has been identified in urine after rye consumption by Bondia-Pons et al.^30^.

**Figure 2:**
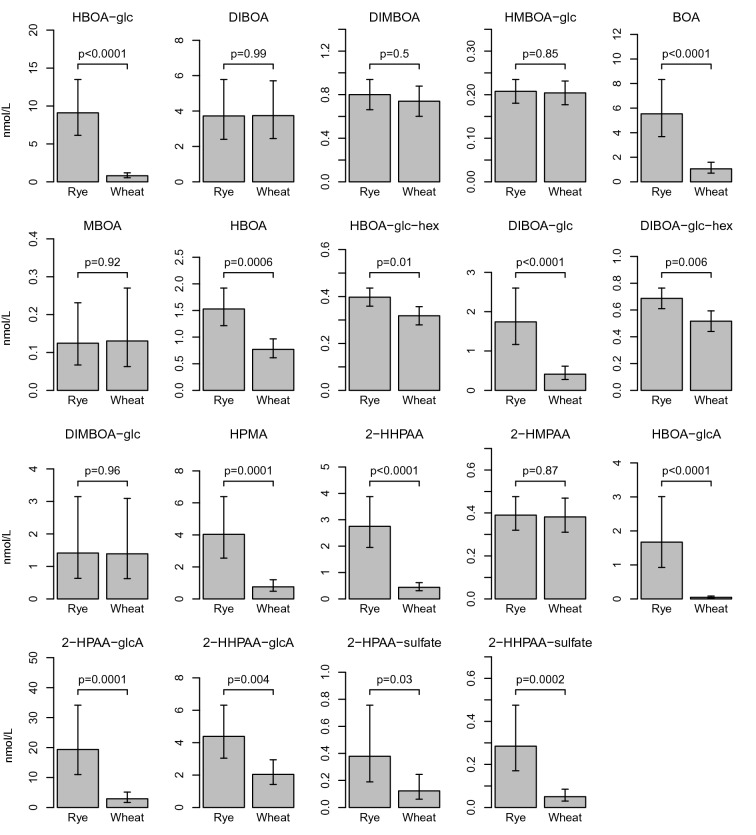
Difference in BX and BX metabolite concentration between the rye and wheat intervention. Data presented as mean and 95% confidence interval.

**Figure 3:**
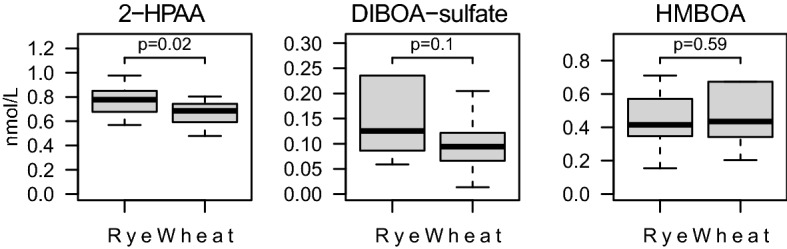
Difference in BX and BX metabolite concentration between the rye and wheat intervention, rank transformed variables, presented as box-and-whisker plots (median, first and third percentiles, range).

**Figure 4:**
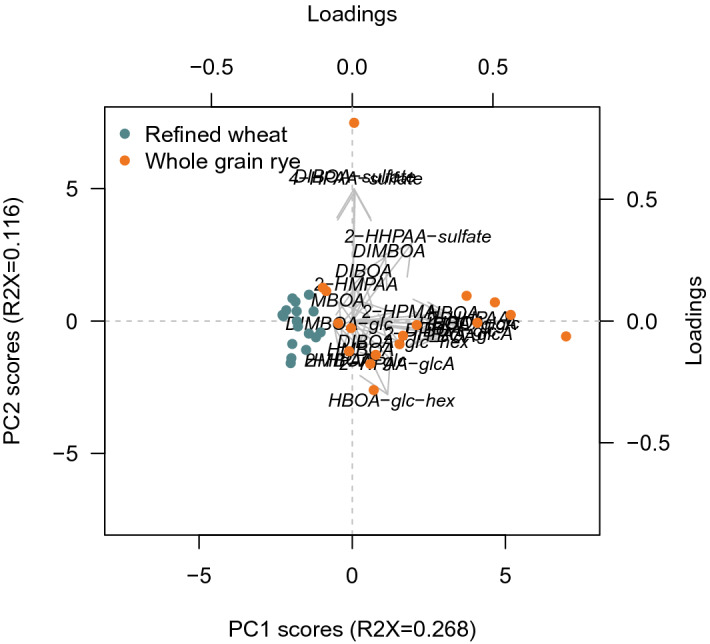
PCA of BX and BX metabolites for the rye and wheat intervention.

The structure of HBOA-glcA was assigned through the comparison of its mass spectrum with the spectra of the pure standards of HBOA and HBOA-glc as seen in Fig. 1, b1, b2 and b3. The fragments characteristic of HBOA (m/z 164/136/118/108) are recognizable in all three spectra, and, similar to DIBOA-glcA, peaks characteristic to glucuronic acid are clearly showed (m/z 193/175/113/85)^36^. Enough material was sufficiently purified for a confirmation of the structure of HBOA-glcA by advanced NMR techniques and comparison to a custom synthesized standard (Supplementary Text 5, Fig. S2 online). Along with HBOA-glcA the sulfate conjugate of HBOA could be expected and was therefore sought for, however, no compound of that supposed structure was detected.

Hanhineva et al.^27^ detected sulfates of 2-HPAA and 2-HHPAA in plasma samples from rye consuming subjects. These findings led us to expect that phase II metabolites of these well-known microbial degradation products of BXs^38,39^ should be present in the urine. The structures of 2-HPAA-glcA and 2-HPAA-sulfate were tentatively elucidated by comparing their mass spectra with the spectrum of 2-HPAA (m/z 150/108) (Fig. 1, c1) and by assuring the presence of the glucuronic acid fragments in 2-HPAA-glcA (Fig. 1, c3), and the HSO_4_^−^ peak (m/z 97) and the neutral loss of 80 corresponding to SO_3_ in 2-HPAA-sulfate (Fig. 1, c4). The retention times and spectra were compared to pure standards of the glucuronide and sulfate of 4-HPAA, which are established metabolites of the painkiller paracetamol and readily commercially available, to ensure correct annotation. The structure for 2-HPAA-glcA was confirmed by advanced NMR techniques (Supplementary Text 5, Fig. S3 online) and subsequently also comparison to a custom synthesized standard. The structures of 2-HHPAA-glcA and 2-HHPAA-sulfate were tentatively elucidated as above described. The 2-HHPAA fragments (m/z 166/148/118/108) could be recognized in both the conjugates’ spectra along with the respective conjugate peaks and neutral losses as seen in Fig. 1, d1, d3 and d4. We were able to confirm the structure of 2-HHPAA-glcA by advanced NMR techniques (Supplementary Text 5, Fig. S4 online).

### Concentrations of BXs and metabolites in plasma from men with indolent prostate cancer after whole grain/bran rye intervention

**Figure 5:**
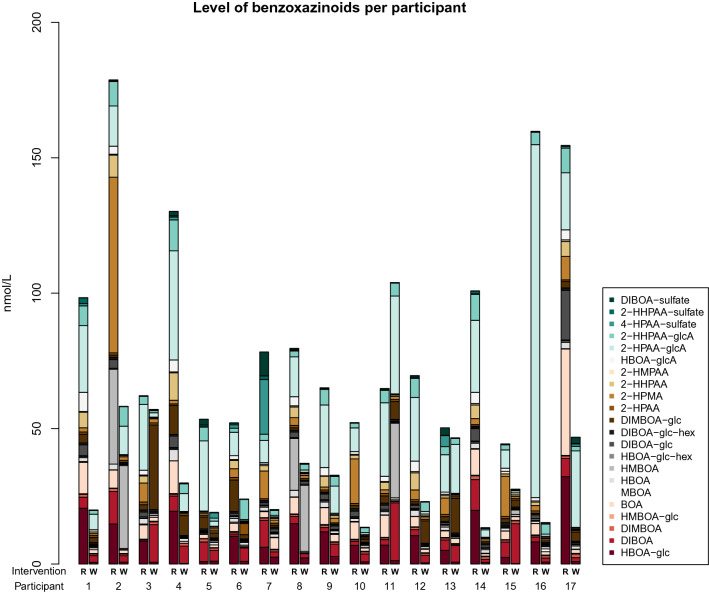
Content of BX and BX metabolites per intervention and participant. R = Whole grain rye/bran, W = Refined wheat.

The established quantitative method for determination of 22 BX metabolites (Supplementary Text 4 online) was applied to samples from the human intervention study to investigate how concentrations were altered in response to whole grain/bran rye intake vs refined wheat control. The concentrations of the BXs 2-HPAA-glcA, HBOA-glc, BOA, 2-HHPAA-glcA, HPMA, 2-HHPAA, DIBOA-glc, HBOA-glcA, HBOA, DIBOA-glc-hex, HBOA-glc-hex, 2-HPAA-sulfate, 2-HPAA and 2-HHPAA-sulfate were significantly higher after 6 week of whole grain/bran rye compared to the wheat intervention (Figs. 2, 3, (0.0001 < *p* ≤ 0.03)), presented in descending order of highest concentration in the whole grain/bran rye intervention. The effect of the interventions on the metabolites is visualized in a PCA in Fig. 4, where the rye intervention clearly separates from the wheat intervention. The variation of the concentration and relative composition of different BXs and their metabolites varied considerably among participants within treatments (Fig. 5).

Our trial included 485 g of intervention products per day. Previous human trails with similar study design have included 404–418 g rye bread per day during 2–3 weeks, containing 64.5–143 µmol BXs per day^28,35^. This corresponded to a daily amount of the BXs BOA 3.6–12.1 µmol, HBOA 0.9–3.1 µmol, MBOA 0.01–15.1 µmol, HBOA-glc 0.5–2.7 µmol, HBOA-glc-hex 6.5–6.8 µmol, HMBOA-glc 0.08–0.1 µmol, DIBOA 3.3–7.7 µmol, DIBOA-glc 25–62.2 µmol, DIBOA-glc-gex 21.7–22.4 µmol and DIMBOA-glc 0.5–1.1 µmol. The concentration of HBOA-glc and DIBOA-glc in our trial are higher compared to what was found in Jensen et al.^28^ but lower compared to the findings in Adhikari et al.^35^, HBOA-glc 4.0–31.7 nmol/L and for DIBOA-glc 0–8.0 nmol/L^28,35^. Other studies have not quantified the level of BXs in food products^12^ or the levels of both BX in food product and/or in plasma are reported as MS/MS fragmentation^27^, nominal mass intensity^26^ or mean difference in peak area^30^, which makes it difficult to relate intake of BXs to concentrations in foods and blood. The intake of rye products was slightly higher in our study compared to Jensen et al.^28^ and Adhikari et al.^35^. The higher amount of rye products in our study together with different type of rye products used, may explain why there are detectable levels of several BXs in our trials and not in Jensen et al.^28^ and Adhikari et al.^35^. It may also explain the difference in plasma concentration of HBOA-glc and DIBOA-glc between the trials. However, more studies with similar study design to elaborate on the level of BX molecules in plasma after certain durations of intake and to establish pharmacokinetic parameters of BX compounds are warranted.

The metabolite at highest concentration after the rye diet was 2-HPAA-glcA, followed by HBOA-glc. Previous animal^29,31^ and human^12,28,30,35^ studies have found that HBOA-glc is the BX found at highest concentration in blood after interventions with rye. In another study^27^, the most abundant BXs in blood after rye intervention were sulphurated 2-HPAA and 2-HHPAA. DIBOA-glc has been reported as the most abundant BX-molecule in rye-based foods such as bread and cereals^29–29,35^. It has been suggested that DIBOA-glc is converted to HBOA-glc upon ingestion^29^ meanwhile 2-HPAA probably is derived from a BOA intermediate^38^, originating from DIBOA^38,40^. The studies with highest concentration of HBOA-glc in plasma have used whole grain rye products^12,28,35^ whereas the study with higher levels of 2-HPAA and 2-HHPAA included wholegrain rye products based on sourdough or bioprocessed rye (enzyme and yeast)^26,27,41^. In our study, participants were supplied with foods containing wholegrain rye with fermented rye bran, i.e. containing both whole grains and added bran. The high concentration of 2-HPAA-glcA is likely related to the fermented rye bran. Food processing have a central role in the concentration and composition of BX molecules due to enzymatic activities or microbial processes^27,28^.

### The associations between circulating BX compounds and total PSA

The total PSA was lower in plasma after intervention with whole grain/bran rye compared to fiber supplemented wheat^14^. We investigated the correlation between PSA and the different measurable BX compounds in the circulation and interestingly, we found that HBOA-glc, HBOA-glcA, 2-HHPAA and 2-HPAA-glcA were significant inverse partially correlated with PSA (Table 2). To our knowledge, no previous investigation has linked BX concentrations to PSA or other biomarkers of prostate cancer. Our results are in line with the findings of the animal^7–9^ and cell line studies^21–24,42^, where an effect of rye or specific BXs had an inhibitory effect on prostate cancer cells. The specific BXs tested were DIBOA and DIMBOA^21–24,42^. Future studies should investigate the effect of an extended spectrum of BX molecules, including the ones correlated with PSA in our study, and preferably on several prostate cancer cell lines. A human pilot study suggested that high intake of rye bran caused apoptosis of prostate tumors, but BX molecules were not analyzed^25^. In another human trial, one-week with whole grain rye consumption in men with prostate cancer resulted in higher BXs concentrations detected in plasma and urine but also in prostate tissue biopsies. Together these results suggest that a high rye intake leads to quantifiable levels of BX in the circulation and in the prostate tissue^12^ and that circulating BX levels are associated with biomarkers of prostate cancer. Often phase II metabolites, i.e. conjugated substances, are assumed to be less biological active, but there are exceptions. For example morphine-6-glucuronide has a higher analgesic effect than the non-glucuronide form^43^. The result from our study suggests that at least some of the conjugated forms may be biologically active or could later have been de-conjugated and distributed further in the organism. Further studies in humans are needed to verify this.

**Table 2:** Partial spearman correlation between total PSA and BX and BX metabolites significant between intervention (whole grain rye/bran and refined wheat), adjusted for id*.

	Correlation	*p*-value
HBOA-glc	− 0.13	0.04
BOA	− 0.10	0.16
HBOA	− 0.06	0.39
HBOA-glc-hex	− 0.08	0.22
DIBOA-glc	− 0.12	0.08
DIBOA-glc-hex	− 0.05	0.49
2-HPAA	0.06	0.43
2-HPMA	− 0.06	0.42
2-HHPAA	− 0.14	0.03
HBOA-glcA	− 0.20	0.01
2-HPAA-glcA	− 0.18	0.004
2-HHPAA-glcA	− 0.07	0.28
2-HPAA-sulfate	− 0.02	0.79
2-HHPAA-sulfate	− 0.03	0.70

*For each variable, number of comparisons of complete data was within the range 178–247.

The fact that rye products treated with different processing techniques result in variations in the content of BXs and BX related molecules creates possibilities for industry to tailor compound profiles by optimizing the processes to bring more healthy foods to the market.

### Limitations and strengths

Our study has some limitations but also strengths. A limitation of this study is the lack of synthetic reference standards for all identified BX compounds which prevents absolute quantification of all compounds. However, we used calibration curves of the most similar metabolites where such were available for semi-quantitation. Another limitation is the lack of analysis of the BXs content in the intervention foods. However, comparing our results to previous studies with similar foods consumed, could give us a rough estimation of the intake. A third limitation is the small sample size, increasing the risk of chance findings. Our results should therefore be interpreted with caution and needs to be verified in independent studies. We acknowledge that rye-based foods contain many other compounds than BX that may be of relevance to prevention/mitigation of prostate cancer, such as lignans^45,46^ and indirect metabolic effects on insulin/C-peptide^44^. Such compounds and processes may exert beneficial effects in parallel with BXs. However, BX has so far only been investigated in a few human studies and was therefore the focus here. A major strength of this study is that we managed to isolate and identify novel BX compounds from urine as well as integrating them in the first quantitative/semiquantitative method for determination of these compounds in human plasma samples. Another strength is the cross over design, were the between-participant variation can be eliminated when evaluating the effect of the diets. The risk for carry over is low, due to short half-life of BXs, shown in the fact there were no differences in concentration between weeks. A third strength is that we were able to quantify many BX and BX-metabolites in plasma for the first time in human plasma and their identities were confirmed by NMR.

## Conclusion

We have successfully established a new method of comprehensive, quantitative analysis of BXs, including compounds not quantified before, in plasma. In total seven BXs and seven BX metabolites were significantly higher in plasma after a diet with whole grain/bran rye foods compared to a diet with refined wheat supplemented with a pure fiber in men with indolent prostate cancer. The BXs HBOA-glc, HBOA-glcA, 2-HHPAA and 2-HPAA-glcA were significantly inversely correlated to the total PSA level, suggesting a potential association with prostate cancer growth. These findings warrant further investigations in larger clinical trials with defined BX intakes.

## Supplementary Information

Supplementary Information 1.

Supplementary Information 2.

## Data Availability

The datasets generated during and/or analyzed during the current study are available from the corresponding author on reasonable request.

## Abbreviations

BXs: Benzoxazinoids
2-HHPAA: 2-Hydroxy-N-(2-hydroxyphenyl)-acetamide
2-HHPAA-glcA: 2-Hydroxy-N-(2-β-D-glucuronopyranosyloxy-phenyl)-acetamide
2-HHPAA-sulfate: 2-(2′-Hydroxyacetamido)-phenyl sulfate
2-HMPAA: N-(2-hydroxy-4-methoxyphenyl)-acetamide
2-HPAA: N-(2-hydroxyphenyl)-acetamide
2-HPAA-glcA: N-(2-glucuronopyranosyloxy-phenyl)-acetamide
2-HPAA-sulphate: 1-Acetamidophenyl sulphate
2-HPMA: N-(2-hydroxyphenyl)-malonamic acid
BOA: Benzoxazolin-2-one
DDA: Data dependent acquisition
DIBOA: 2,4-Dihydroxy-1,4-benzoxazin-3-one
DIBOA-glc: 2-β-D-glucopyranosyloxy-4-hydroxy-1,4-benzoxazin-3-one
DIBOA-glc-hex: 2-[4-O-hexosepyranosyl-β-D-glucopyranosyl]-oxy-4-hydroxy-1,4-benzoxazin-3-one
DIBOA-sulfate: 4-Hydroxy-1,4-benzoxazin-3-one-2-yl sulfate
DIMBOA: 2,4-Dihydroxy-7-methoxy-1,4-benzoxazin-3-one
DIMBOA-glc: 2-β-D-glucopyranosyloxy-4-hydroxy-7-methoxy-1,4-benzoxazin-3-one
EMS: Enhanced mass scan
EPI: Enhanced product ion scan
HBOA: 2-Hydroxy-1,4-benzoxazin-3-one
HBOA-glc: 2-β-D-glucopyranosyloxy-1,4-benzoxazin-3-one
HBOA-glcA: 2-Glucuronopyranosyloxy-1,4-benzoxazin-3-one
HBOA-glc-hex: 2-[4-O-hexosepyranosyl-β-D-glucopyranosyl]-oxy-1,4-benzoxazin-3-one
HMBOA: 2-Hydroxy-7-methoxy-1,4-benzoxazin-3-one
HMBOA-glc: 2-β-D-glucopyranosyloxy-7-methoxy-1,4-benzoxazin-3-one
MBOA: 6-Methoxybenzoxazolin-2-one
MRM: Multiple reaction monitoring
PSA: Prostate specific antigen
