# Supplementary material for: An inverse association between plasma benzoxazinoid metabolites and PSA after rye intake in men with prostate cancer revealed with a new method
Source: Sci Rep. 2022 Mar 28;12:5260. doi: 10.1038/s41598-022-08856-z (PMC8960836; doi:10.1038/s41598-022-08856-z)
Supplement: Supplementary file 1 — Supplementary Information 1. [file 41598_2022_8856_MOESM1_ESM.pdf]

Supplementary materials

An inverse association between plasma benzoxazinoid metabolites and PSA after rye intake in men with prostate cancer revealed with a new method

Elise Nordin[1#], Stine K. Steffensen[2#], Bente B. Laursen[2], Sven-Olof Andersson[3], Jan-Erik Johansson[3], Per Åman[4], Göran Hallmans[5], Michael Borre[6], Dan Stærk[7], Kati Hanhineva[1,8,9] Inge S. Fomsgaard[2 ¤], Rikard Landberg[1,5¤]

[1] Division of Food and Nutrition Science, Department of Biology and Biological Engineering, Chalmers University of Technology, Gothenburg, Sweden

[2] Department of Agroecology, Aarhus University, Forsøgsvej 1, DK-4200 Slagelse

[3] Department of Urology, Faculty of Medicine and Health, Örebro University, Örebro, Sweden.

[4] Swedish University of Agricultural Sciences, Department of Molecular Sciences, Box, 7015, Uppsala, Sweden.

[5] Department of Public Health and Clinical Medicine, Umeå University, Umeå, Sweden.

[6] Department of Urology, Aarhus University Hospital, Palle Juul-Jensens Boulevard 99, 8200 Aarhus N, Denmark

[7] Department of Drug Design and Pharmacology, Faculty of Health and Medical Sciences, University of Copenhagen, Universitetsparken 2, DK-2100 Copenhagen, Denmark

[8] Department of Life Technologies, Food Chemistry and Food Development unit, FI-20520 Turku, Finland

[9] University of Eastern Finland, School of Medicine, Institute of Public Health and Clinical Nutrition, FI-70210 Kuopio, Finland

# Contributed equally, corresponding author
¤ Contributed equally, corresponding author

Supplementary Text 1.

Discovery of BX-related metabolites from urine samples with high BX concentration using Data Dependent Acquisition Mass Spectrometric (DDA-MS)

The LC-MS system consisted of an Agilent 1260 infinity HPLC system with a quaternary pump (Santa Clara, CA, USA) coupled to an AB Sciex 4500 triple-quadrupole trap mass spectrometer (QTRAP/MS) (AB Sciex, Framingham, USA) with electrospray ionization (ESI). The autosampler was fitted with a cooler set at 10 °C. The column oven was set at 30 °C and equipped with a Synergi Polar column and pre-column (4 µm, 2.1*250 mm, Phenomenex). The gradient was run as a binary gradient using a weak eluent (A) consisting of 7% acetonitrile and 20mM acetic acid in water; and a strong eluent (B) consisting of 78% acetonitrile and 20 mM acetic acid in water.

Nitrogen gas was used as a collision gas to generate MS/MS fragmentations. Instrument-dependent parameters for the mass spectrometer were as follows: Curtain gas, 35 psi; ion spray voltage, -4300 V; source temperature, 450 °C; gas 1, 90 psi; gas 2, 90 psi; the collision gas (CAD) was set to -2. The chromatographic method was run at 300 µl/min for 23 min with an injection volume of 10.0 µL. The gradient was as follows: At 0 min A/B was 100/0; at 1 min A/B: 92/8; at 3 mins A/B: 90/10; at 13 min A/B:30/70; at 14 min A/B:10/90; at 16 min A/B:10/90; at 17 min A/B:100/0; and end at 23 min in A/B:100/0.

Two DDA-MS methods were set up, an untargeted and a targeted method. The untargeted DDA-MS method (EMS-EPI) was set up combining a full scan (EMS, m/z 200-550 Da) as survey scan, with a product ion scan (EPI, *m*/*z* 50-500 Da) as dependent scan, both utilizing the enhanced sensitivity of the linear ion trap. Similarly, a targeted DDA-MS method (MRM-EPI) was set up utilizing known fragmentation patterns of BX-standards and non-BX commercially available phase 2 glucuronide and sulfate metabolites (Group 4 compounds). The method took advantage of the high sensitivity of the triple quadrupole using targeted ion reaction mass transitions (Table 1, manuscript) as survey scan, combined with the EPI scan (*m*/*z* 50-500 Da) as dependent scan. Both the targeted and untargeted DDA-MS methods were set up with the purpose of obtaining spectral data as good as possible (cycle time 2.5 sec and general peak width at half height 15 sec). Further settings were based on the work published by Bhattarai et al (2021)[1].

Supplementary Text 2.

Purification of BX-related metabolites from urine rich in BX from a human pilot study

Some unidentified signals present only after the rye intervention and with BX-like MS fragmentation patterns, determined by the data dependent acquisition-based MS-methods (DDA-MS), were marked as interesting for further investigation. Available BX-related standards (See Table 1, group 1 and 2 (manuscript)) were used to build the methods and interpret the data. Most of the interesting signals could be recognized as probably being a known aglucone coupled to a glucuronic acid or sulfate i.e. common phase II metabolites. The combined urine taken after the intervention was subjected to subsequent solid phase extractions and DDA-MS was used for fraction selection with the purpose to isolate the compounds of interest sufficiently for identification by NMR. Fractions with a content of BX related metabolites as determined by the DDA-MS analysis, where mass spectra of BX-standards were compared to spectra of unknown

peaks, were subjected to further SPE separation this time using weak anion exchange SPE (WAX), utilizing the inherent anionic properties of glucuronic acid and sulphate derivatives. Prior to passing the reconstituted sample through the WAX, the sample was passed through a strong cation exchange cartridge (MCX). This allowed for the removal of basic and twitter ionic compounds by binding to the strong cationic exchange before selective and reversable binding of anionic compounds to the weak anion exchange column. Meanwhile neutral, polar compounds were washed away due to poor interaction with the stationary phases. Hippuric acid, endogenic to urine, was a major obstacle in the cleaning process as it was present in very high concentrations and had acidic properties like the glucuronic acid derivates. Hence, method development also focused on conditions that would separate the BX-derivative from hippuric acid as much as possible. This was obtained to a large extent by eluding the weak acid metabolites first with consecutive small fractions of 2% hydrochloric acid and 20% methanol in water and afterwards 2% hydrochloric acid and 40% methanol in water leading to the BX-glucuronides eluding in the first fractions and hippuric acid in the later. The BX-sulfates were retained until eluted by base. In this way a good separation into metabolite categories was obtained prior to moving on to semi-preparative HPLC. However, HPLC-UV revealed that the fractions still contained a lot of endogenic urine metabolites and that the BX-metabolites were only minor constituents in the fractions. Fractions were collected by 30 seconds time windows and all fractions were analyzed using the DDA-MS methods. In this way, the small peaks corresponding to interesting BX-metabolites were identified and collected. A second round of semi-preparative HPLC-UV using a column with a different selectivity afforded the purified standards. Their structures were primarily assigned by mass spectrometry comparing spectra to that of standard compounds of similar structures.

Supplementary Text 3.

NMR Experiments

NMR spectra were acquired in DMSO-d_6 at 300 K using a Bruker Avance III NMR spectrometer (^{1}H resonance frequency of 600.13 MHz), and ^{1}H and ^{13}C NMR chemical shifts were referenced to the residual solvent signal of DMSO-d_6 (δ_H = 2.50 ppm, δ_C = 39.52 ppm). ^{1}H NMR spectra were recorded with a spectral width of 20 ppm using 30° pulses and 65 k data points. For the 2D NMR experiments, phase-sensitive DQF-COSY and ROESY spectra were recorded using gradient-based pulse sequences with 9 ppm spectral width and 2 k × 512 data points (processed with forward linear prediction to 1 k data points in F1); multiplicity-edited HSQC spectra were acquired with the following parameters: $1J_{C,H}$ = 145 Hz, spectral width 12 ppm for ^{1}H and 140 ppm for ^{13}C, 1730 × 192 data points (processed with forward linear prediction to 512 data points and zero-filling to 1 k data points in F1), and 1.0 s relaxation delay; low-pass filtered HMBC experiments were optimized for $^nJ_{C,H}$ = 8.0 Hz (long-range), $^1J_{C,H}$ min = 125 Hz, and $^1J_{C,H}$ max = 160 Hz and recorded using a spectral width of 12 ppm for ^{1}H and 180 ppm for ^{13}C , 2k × 256 data points (processed with forward linear prediction to 512 data points and zero-filling to 1 k data points in F1), and 1.0 s relaxation delay.

Figure S1 (electronic ppt, zoomable version of Figure 1 in manuscript). Mass spectra of standards (name in red) of DIBOA, DIBOA-glc, HBOA, HBOA-glc, 2-HPAA and 2-HHPAA; and the tentatively assigned phase 2 metabolites DIBOA-glcA, DIBOA-sulfat, HBOA-glcA*, 2-HPAA-glcA*, 2-HPAA-sulfat, 2-HHPAA-glcA*, and 2-HHPAA-sulfat.

*Structure subsequently confirmed by NMR and comparison to custom synthesized standards

Supplementary Text 4.

Determination of recovery and limit of detection (LOD) of BX compounds in plasma

To obtain a plasma matrix that contained no BX compounds for spiking experiments, blood from pigs that had only been fed with refined wheat for several weeks was used. Four standard mixtures (A, C, L, and HH, see Table 1 in manuscript) were spiked into aliquoted pig plasma samples at a concentration of 5 ng/ml plasma in six replicates giving a total of twenty-four recovery samples. The spiked samples were stored together with the original samples at -80 ºC and subsequently thawed, prepared, and analyzed one set of four recovery samples together with each of the six batches of study plasma samples. The mean and the standard deviation of the recovery samples of all six batches were calculated and LOD was determined as 3 x STDDEV of six replicates. For the semi-quantified compounds, no recovery or LOD were determined).

Validation of a quantitative method for determination of BXs compounds/metabolites in plasma

Guidelines for validation of analytical methods aimed at being used for quantification of natural bioactive compounds in food or in consumer's plasma do not exist. Validation guidelines are generally dedicated to methods that are used for analysis of potential toxic contamination (pesticide residues in food)[2]. or for analysis that are meant to assure that then content of a pharmaceutical drug is accurate[3]. It is however a general recommendation that recovery and limit of detection are relevant parameters to include when an analytical ad hoc method is used[4]. The main goal of a method validation is to assure that the method is fit for the purpose. Recoveries between 70 and 120% are often mentioned as acceptable[2]. For seven of the compounds the recovery values are outside this range; however, for six of those compounds the relative standard deviation is <20%, which makes the deviation from recommended recovery range acceptable.

The BX-phase II metabolites semi-purified from urine were semi-quantified in the plasma samples against standard curves of the standards 4-HPAA-glcA and 4-HPAA-sulfate respectively. However, between-sample statistical comparison still provides meaningful interpretations because the inter-sample variation in matrix effect can be disregarded as was found in the work of Steffensen et al.[5] Likewise, a LOD could not be established for the BX-phase II metabolites. In general, LOD varies considerably between compounds and is affected by many factors both of chromatographic properties and mass spectrometric properties. The relatively low and insignificant concentrations found in the plasma samples of some of the BX-phase II metabolites might simply reflect a poor LOD. The recovery estimated from spiked controls, analyzed with all batches are shown below In Table S1 together with the LOD.

Table S1. Recovery percentage and LOD

compound	% Recovery ; mean of all batches	% Recovery; std dev of all batches	LOD ng/ml plasma
HBOA-glc	163	13	0.03

DIBOA	64	21	0.09
HMBOA-glc	122	12	0.01
BOA	123	17	0.1
MBOA	107	14	0.03
HBOA	90	18	0.2
HMBOA	64	18	0.4
HBOA-glc-hex	135	37	0.9
DIBOA-glc	105	21	0.02
DIBOA-glc-hex	98	29	0.09
DIMBOA-glc	77	31	2.8
2-HPAA	101	29	0.03
HPMA	129	17	0.05
2-HHPAA	114	22	0.03
HMPAA	105	18	0.03

Supplementary Text 5.

Structure elucidation of BX metabolites from urine samples

The compound (**Figure S2**), which by MS was suggested to be **HBOA-glcA**, was identified in an inseparable mixture. The HBOA skeleton was identified from the signals for H-5 at δ 6.94 (d, $J_{H5,H6}$ = 7.6 Hz, δ_C 115.4), H-6 at δ 7.00 (t, $J_{H5,H6}$ = $J_{H6,H7}$ = 7.5 Hz, δ_C 122.8), H-7 at δ 6.97 (t, $J_{H6,H7}$ = $J_{H7,H8}$ = 7.5 Hz, δ_C 123.0), H-8 at δ 7.04 (d, $J_{H5,H6}$ = 7.6 Hz, δ_C 117.3), and H-2 at δ 5.59 (s, δ_C 91.1) (see **Spectra 1** and **2**), by comparison with the same region of the spectrum of HBOA-Glc as reference compound.

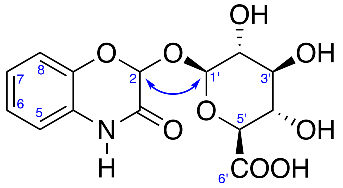

Figure S2. Structure and diagnostic correlation observed in the ROESY spectrum of compound **HBOA-glcA.**

The COSY spectrum (**Spectrum 3**) revealed signals for H-1' at δ 4.70 (d, $J_{H1',H2'}$ = 7.8 Hz, δ_C 97.9), H-2' at δ 2.95 (m, δ_C 72.2), H-3' at δ 3.21 (m, δ_C 75.5), H-3' at δ 3.35 (m, δ_C 71.0), and H-5' at δ 3.88 (d, $J_{H4',H5'}$ = 9.7 Hz, δ_C 75.2). However, whereas the spectra of the reference compound HBOA-Glc showed a complex doublet of doublet of doublet for H-5' and signals from the diastereotopic proton pairs H-6'A and H-6'B, the signal for H-5' was reduced to the above-mentioned doublet and the signal for H-6'A and H-6'B absent, in agreement with a glucuronic acid moiety in **HBOA-glcA** instead of the glucoside unit observed in HBOA-Glc. These observed resonance signals are also in agreement with the resonance signals seen for the glucuronic acid in **2-HPAA-glcA**, *vide infra*. A ROESY correlation (**Spectrum 4** and **Figure S2**) between H-1' and H-2 finally showed the glucuronic acid moiety to be positioned at C-2 of the HBOA skeleton. The compound annotated as **HBOA-glcA** is thus 2-(2*H*-benzo[*b*][1,4]oxazin-3(4*H*)-one-2-yl) β-D-glucopyranosiduronic acid.

The compound annotated as 2-HPAA-glcA (**Figure S3**) was obtained as an inseparable mixture, evident from the complex ^{1}H NMR spectrum with signals of varying intensity (**Spectrum 5**). Starting with the broad unresolved signal for H-3 at δ 8.11, which is down field shifted compared to H-3 in *N*-(2-hydroxyphenyl)acetamid (2-HPAA) where the carbonyl group is hydrogen-bonded with the phenol OH, the COSY spectrum (**Spectrum 6**) revealed correlations between H-3, the overlapping H-4 and H-5 at δ 7.02, and H-6 at δ 7.10.

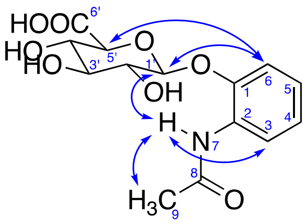

Figure S3. Structure and correlations observed in the ROESY spectrum of compound **2-HPAA-glcA.**

The characteristic singlet signal at δ 9.08 did not display any one-bond CH correlations in the HSQC spectrum (**Spectrum 7**), and was attributed to the amide N-H (similar signal observed for 2-HPAA). Similarly, a methyl singlet was observed at δ 2.09, which together with the above-mentioned signals showed the presence of the 2-HPAA moiety. For the glucuronic acid moiety, doublet signals for H-1' (δ 4.70, d $J_{H1',H2'}$ = 7.7 Hz) and H-5' (δ 3.86, d $J_{H4',H5'}$ = 9.5 Hz) showed COSY cross peaks to H2' at δ 3.39 and H4' at δ 3.44, respectively, the two latter masked by a large water signal. However, also the HSQC spectrum clearly showed H-2' at δ 3.39 ($\delta_{C2'}$ 72.7) and H-4' at δ 3.44 ($\delta_{C4'}$ 71.1) in addition to H-3' at δ 3.33 ($\delta_{C3'}$ 74.8). The connection of the glucuronic acid at C-1 of the 2-HPAA moiety was confirmed by correlations observed between the amide proton H-3 and H-1', H-3, and the methyl group in the ROESY spectrum (**Spectrum 8**) as well as between H-6 of the 2-HPAA moiety and H-1' and H-5' of the glucuronic acid (**see Figure S3**). Finally, a HMBC correlation from H-5' to a signal at δ_C 169.7 confirmed C-6' to be a carboxylic acid (**Spectrum 9**). The compound annotated as **2-HPAA-glcA** is thus 2-(acetylamino)phenyl β-D-glucopyranosiduronic acid and thereby the proposed structure is confirmed.

The compound (**Figure S4**), which by MS was suggested to be **2-HHPAA-glcA**, was also identified in an inseparable mixture. The ^{1}H NMR spectrum of **2-HHPAA-glcA** (**Spectrum 10**) showed the same overall pattern of a 1,2-disubstituted benzene ring as observed for **2-HPAA-glcA**, with H-3 at δ 8.22 (d, $J_{H3,H4}$ = 7.3 Hz, δ_C 119.6), H-4 at δ 7.02 (m, δ_C 122.3), H-5 at δ 7.05 (m, δ_C 123.6), and H-6 at δ 7.65 (d, $J_{H5,H6}$ = 7.8 Hz, δ_C 122.1) as well as the amide singlet resonance at δ 9.28.

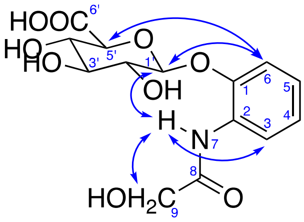

Figure S4. Structure and correlations observed in the ROESY spectrum of compound **2-HHPAA-glcA**

However, instead of the methyl singlet observed at δ 2.09 in the spectrum of **2-HPAA-glcA**, a downfield shifted methylene singlet for H-9 was observed at δ 3.99 (δ_C 61.5), showing a 2-HHPAA skeleton rather than the 2-HPAA skeleton. The HSQC spectrum (**Spectrum 11**) revealed the same pattern for a glucuronic acid for **2-HHPAA-glcA** as seen for **2-HPAA-glcA**, and as seen in **Figure S4**, the same ROESY correlations (**Spectrum 12**) as seen for **2-HPAA-glcA** was observed for **2-HHPAA-glcA**. The compound annotated as **2-HHPAA-glcA** is thus 2-(hydroxyacetylamino)phenyl β-D-glucopyranosiduronic acid.

Spectrum 1. ^{1}H NMR spectrum of **HBOA-glcA** in DMSO-d_6 (600 MHz)

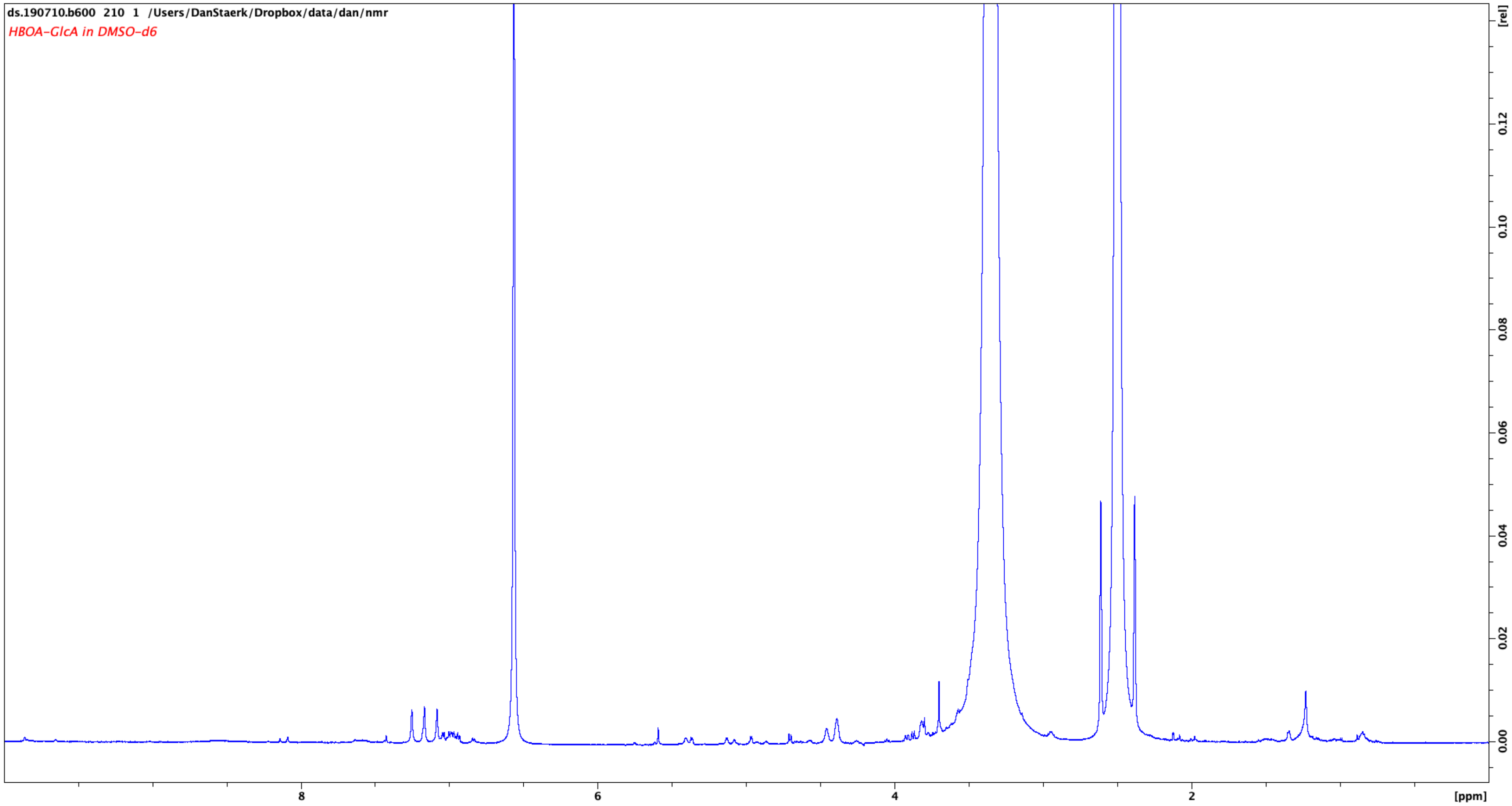

Spectrum 2. HSQC spectrum of **HBOA-glcA** in DMSO-d_6 (600 MHz)

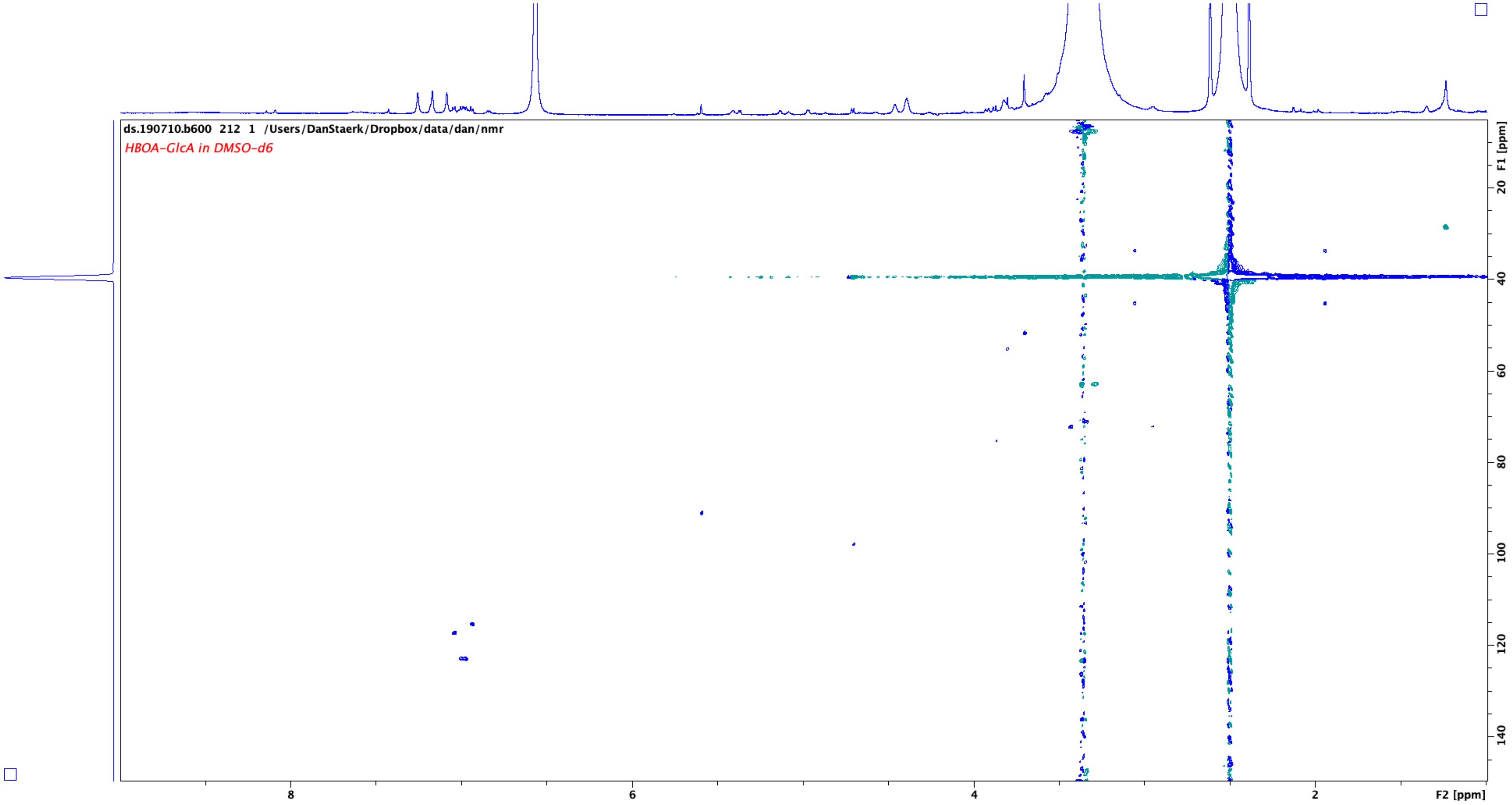

Spectrum 3. COSY spectrum of **HBOA-glcA** in DMSO-d_6 (600 MHz)

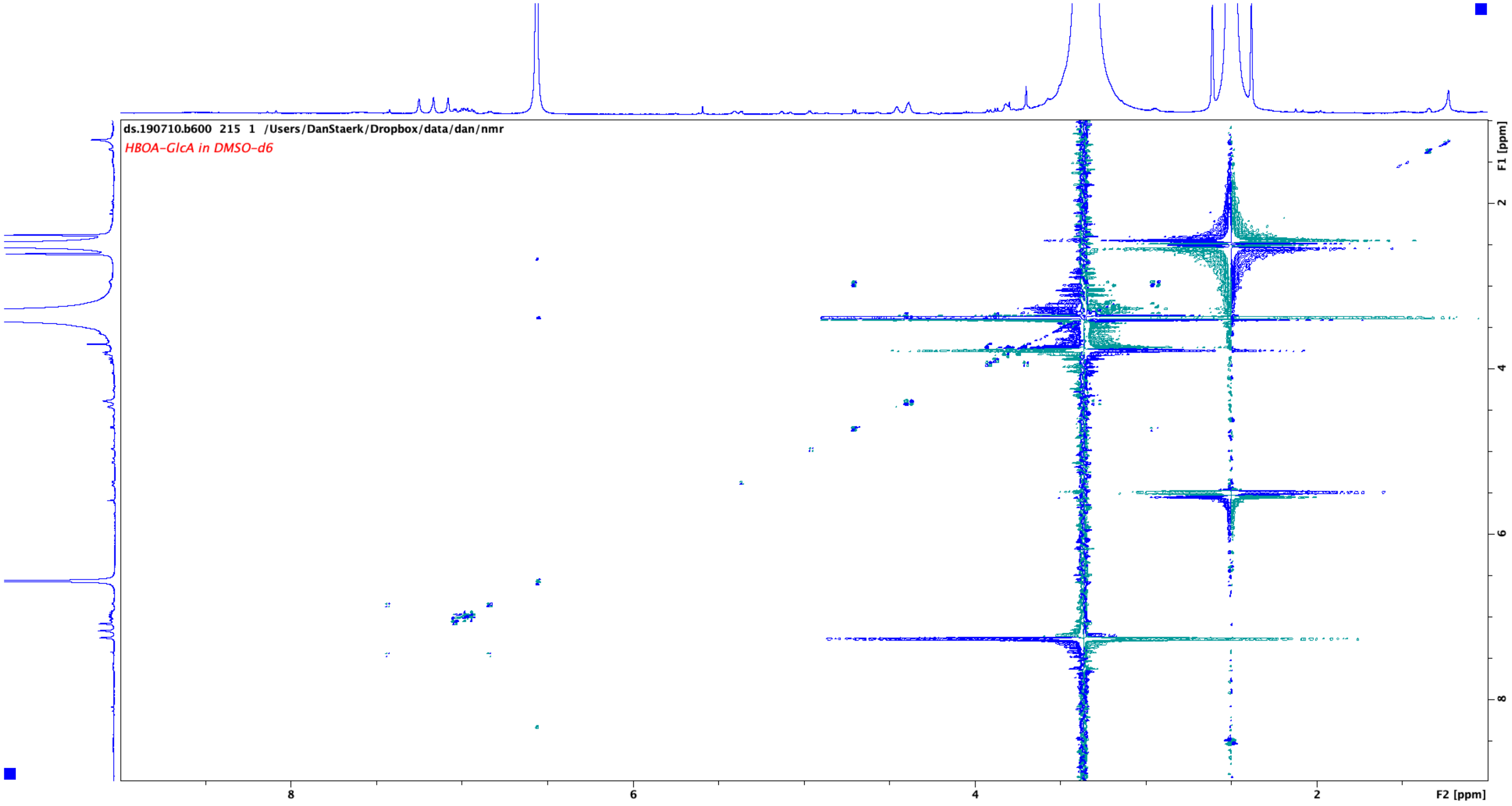

Spectrum 4. ROESY spectrum of **HBOA-glcA** in DMSO-d_6 (600 MHz)

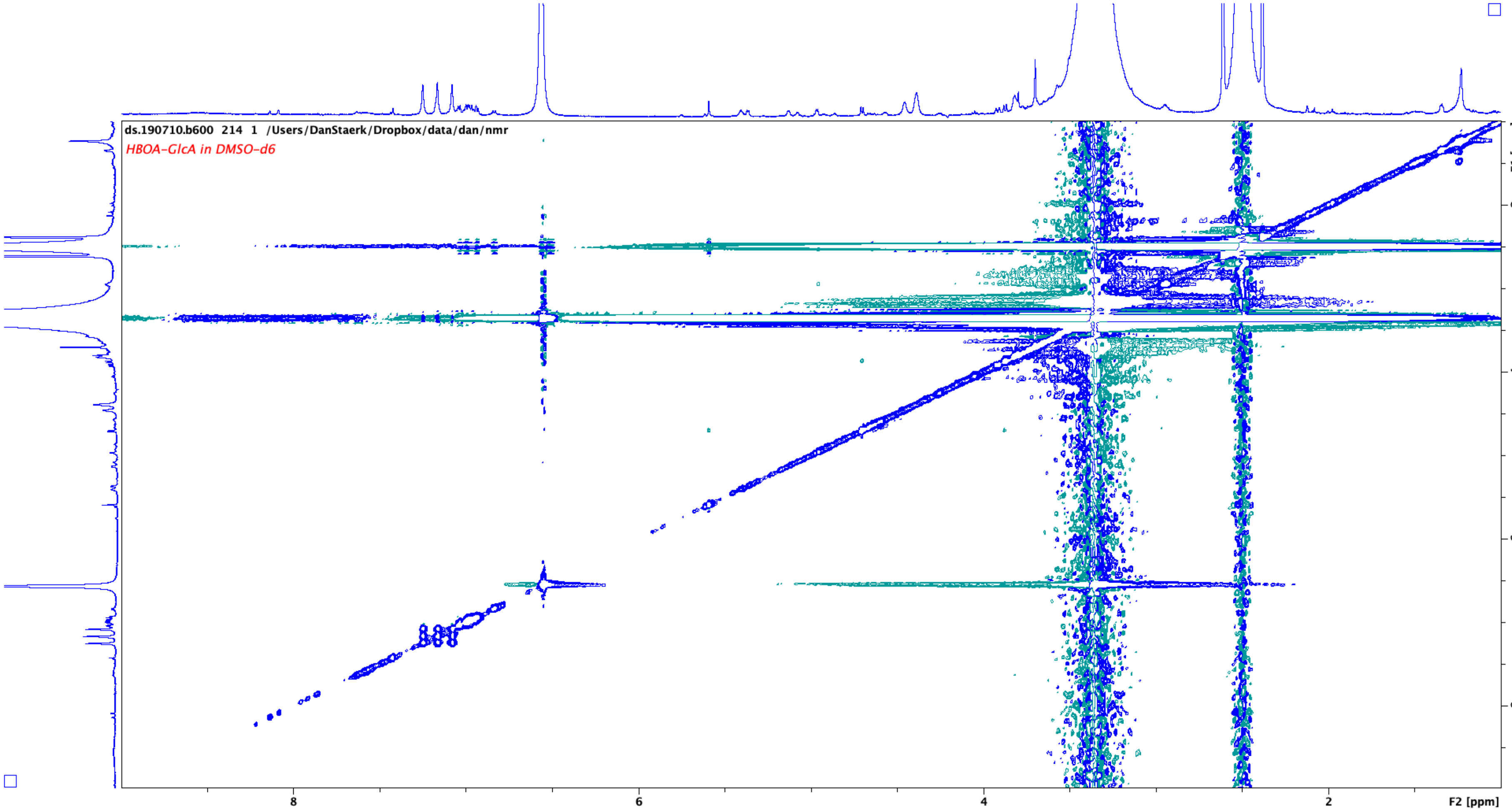

Spectrum 5. ^{1}H NMR spectrum of **2-HPAA-glcA** in DMSO-d_6 (600 MHz)

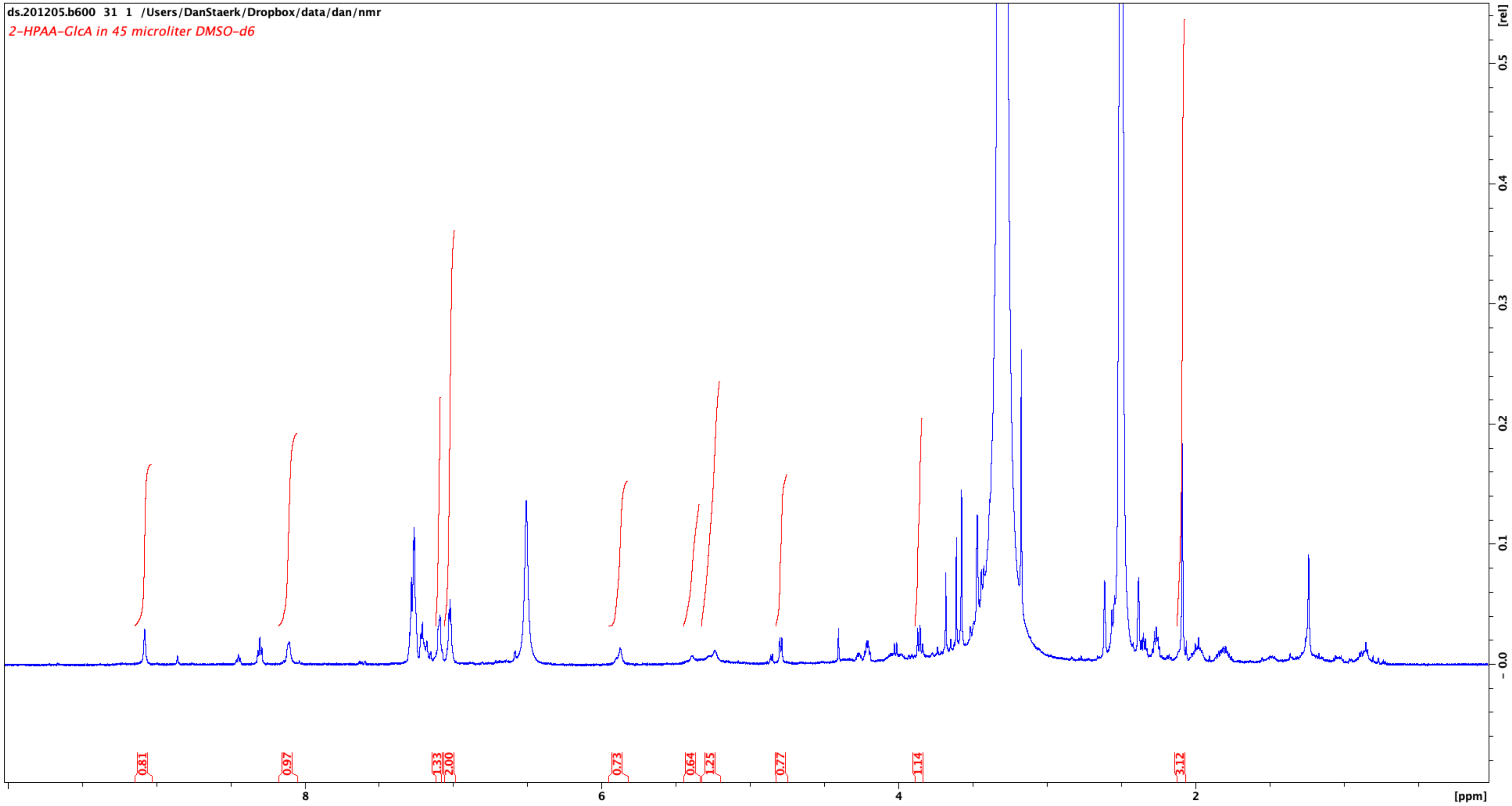

Spectrum 6. COSY spectrum of **2-HPAA-glcA** in DMSO-d_6 (600 MHz)

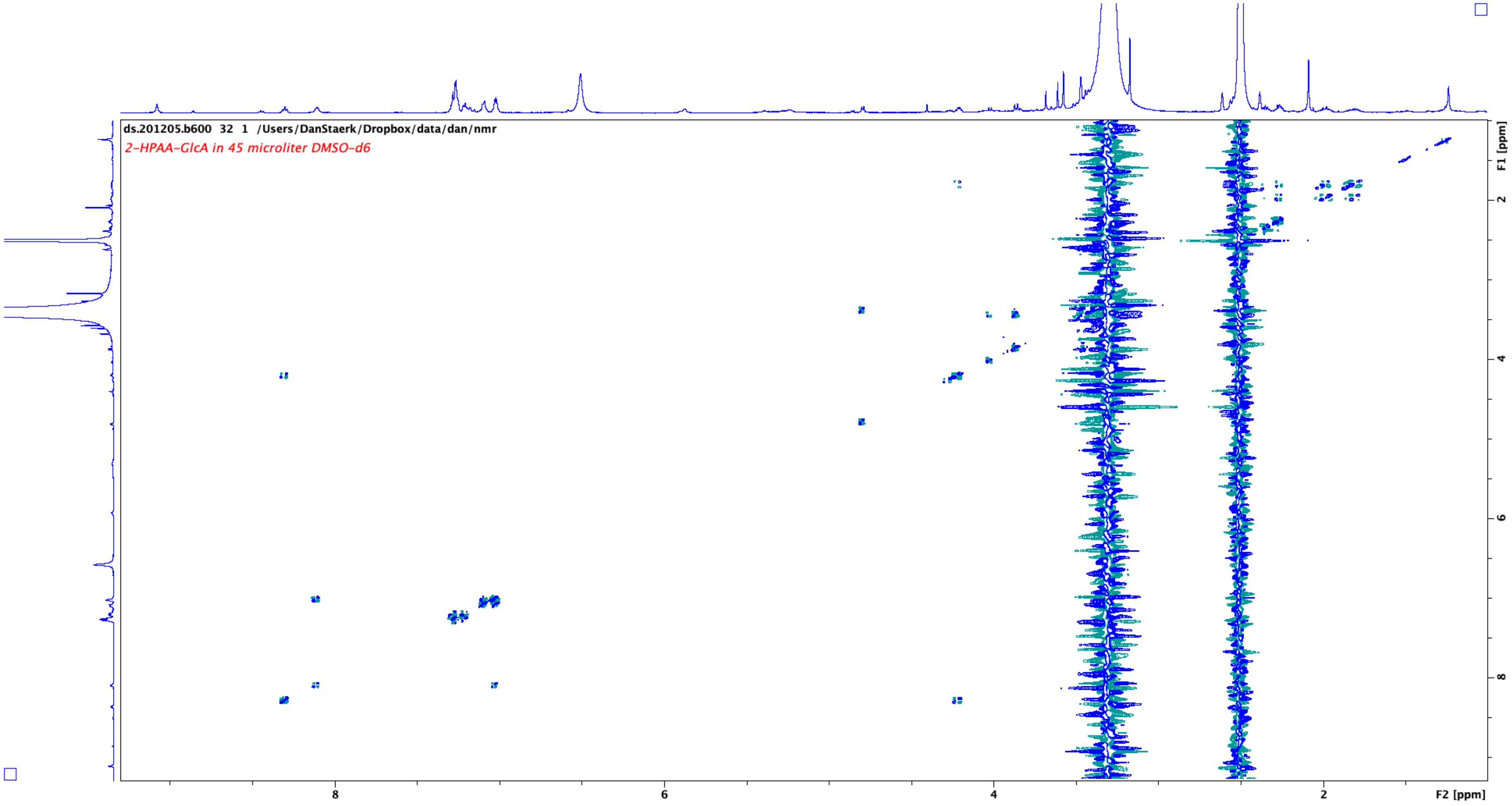

Spectrum 7. HSQC spectrum of **2-HPAA-glcA** in DMSO-d_6 (600 MHz)

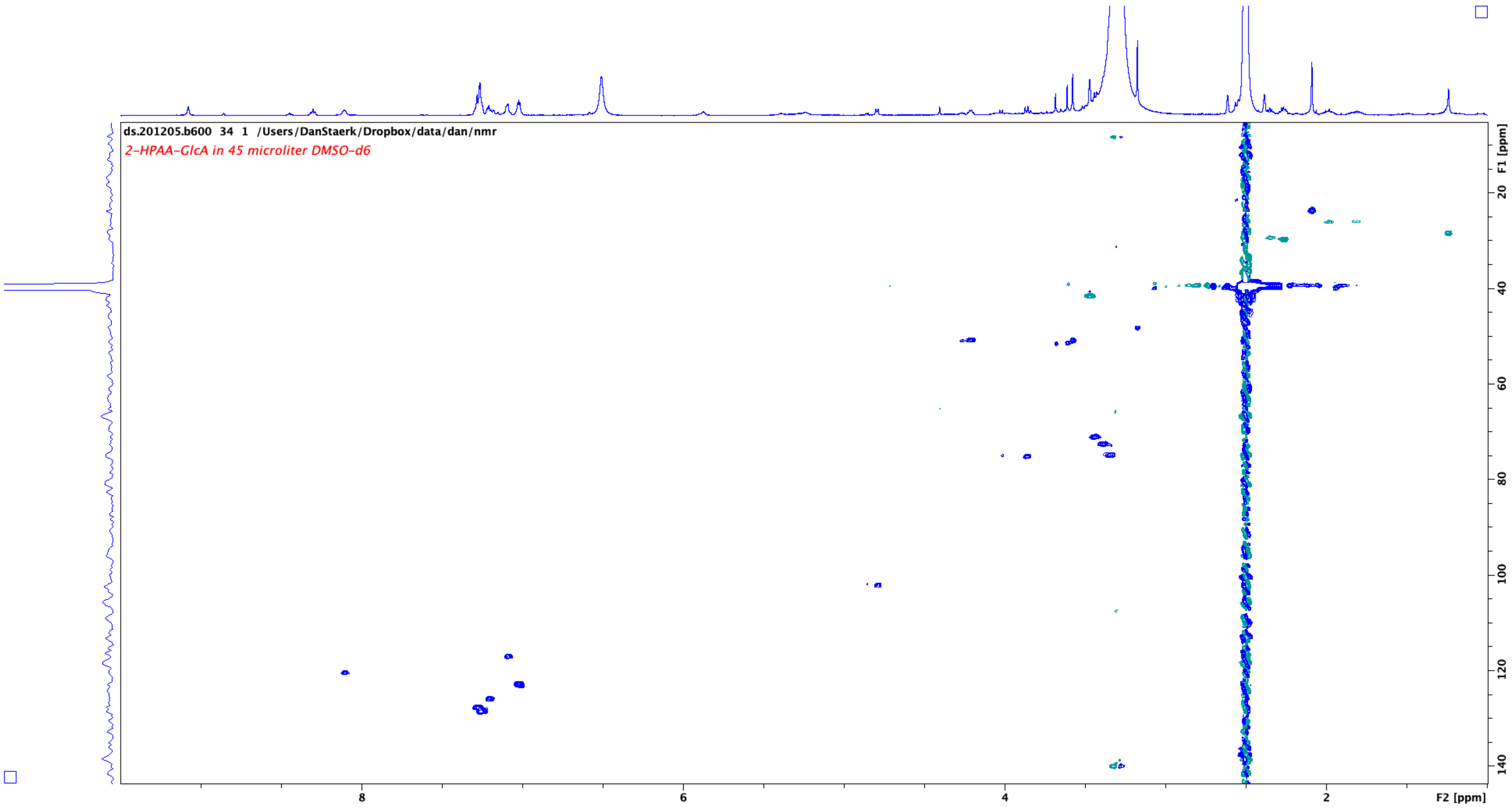

Spectrum 8. ROESY spectrum of **2-HPAA-glcA** in DMSO-d_6 (600 MHz)

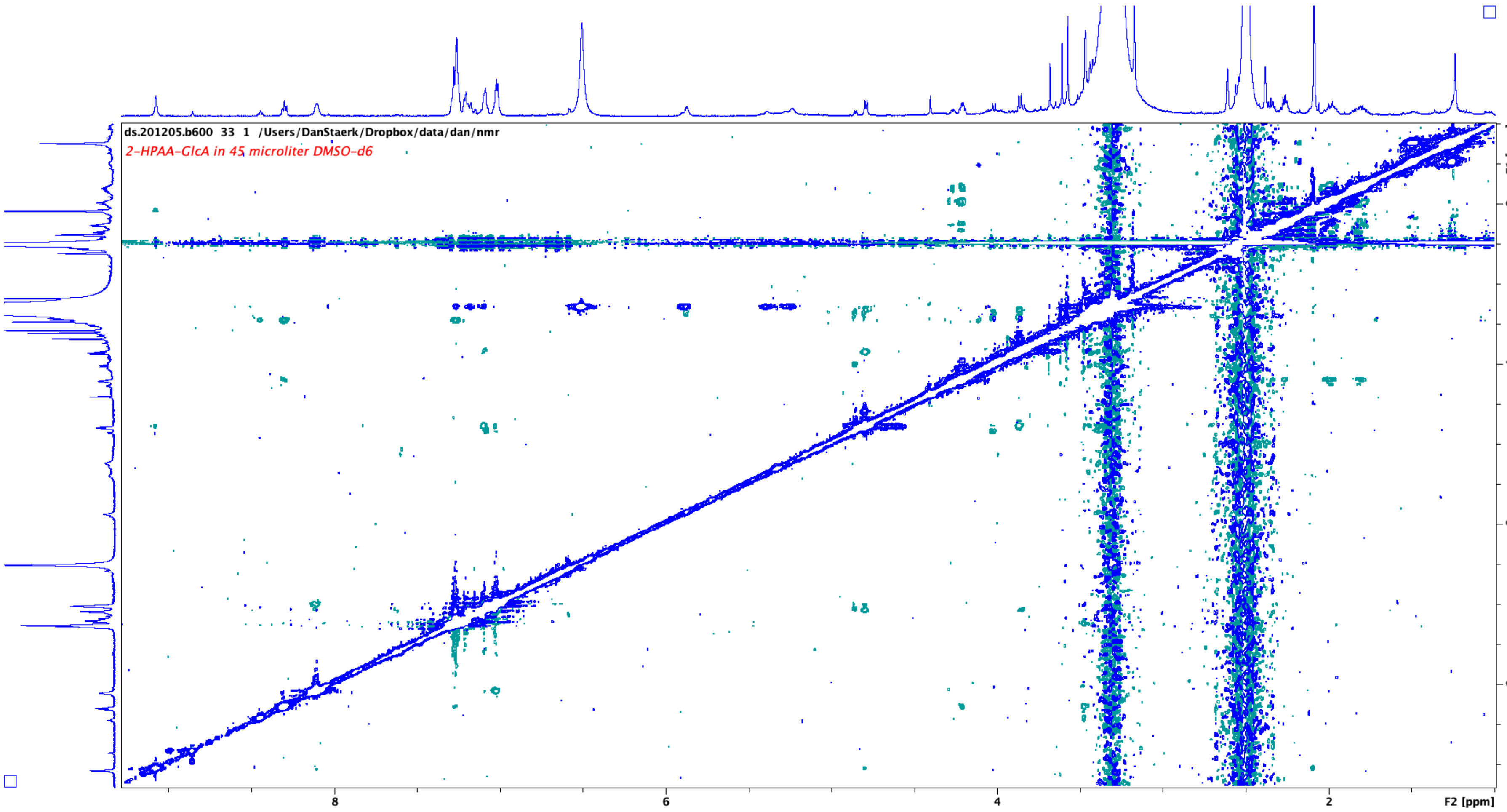

Spectrum 9. HMBC spectrum of **2-HPAA-glcA** in DMSO-d_6 (600 MHz)

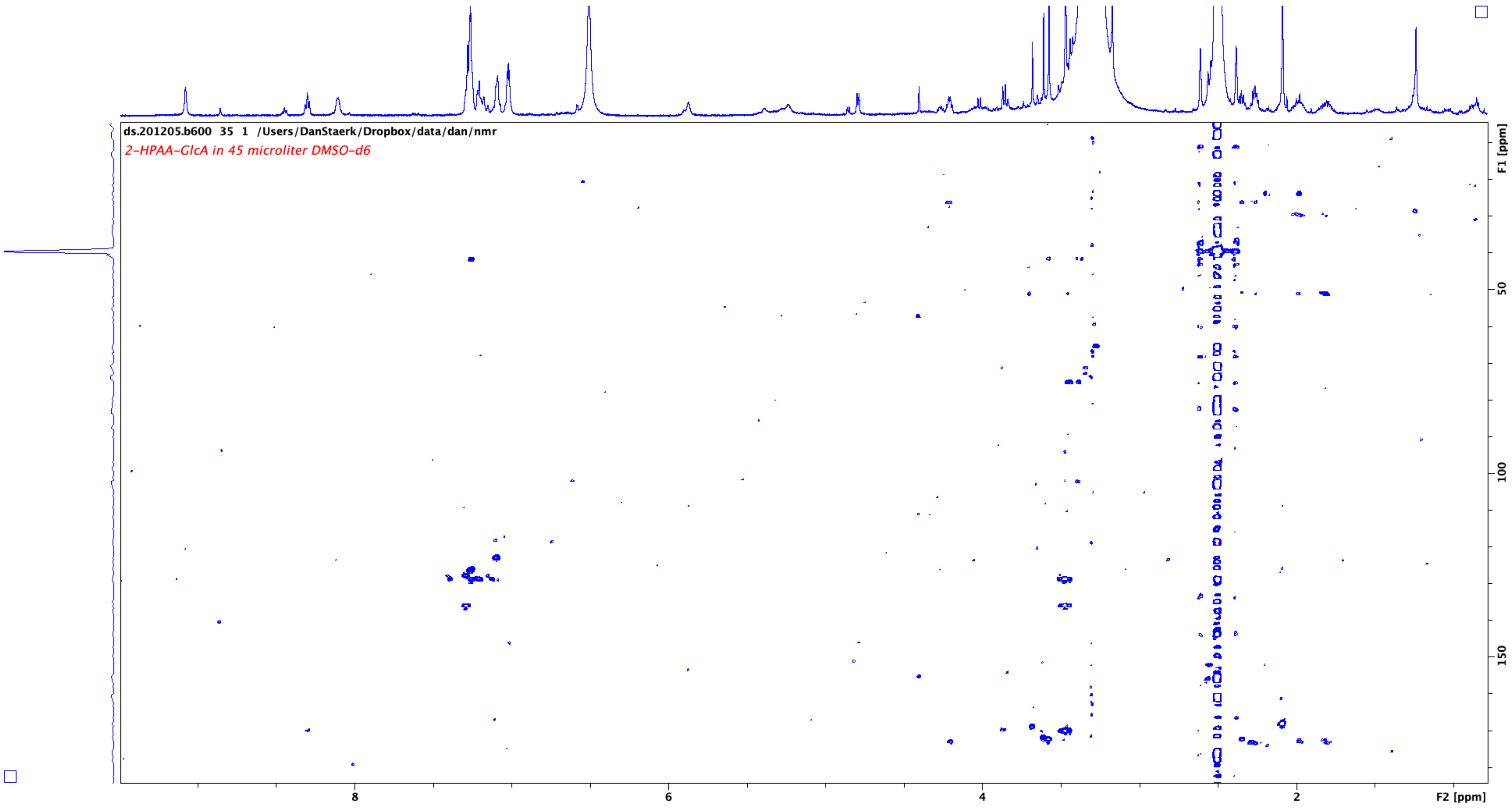

Spectrum 10. ^{1}H NMR spectrum of **2-HHPAA-glcA** in DMSO-d_6 (600 MHz)

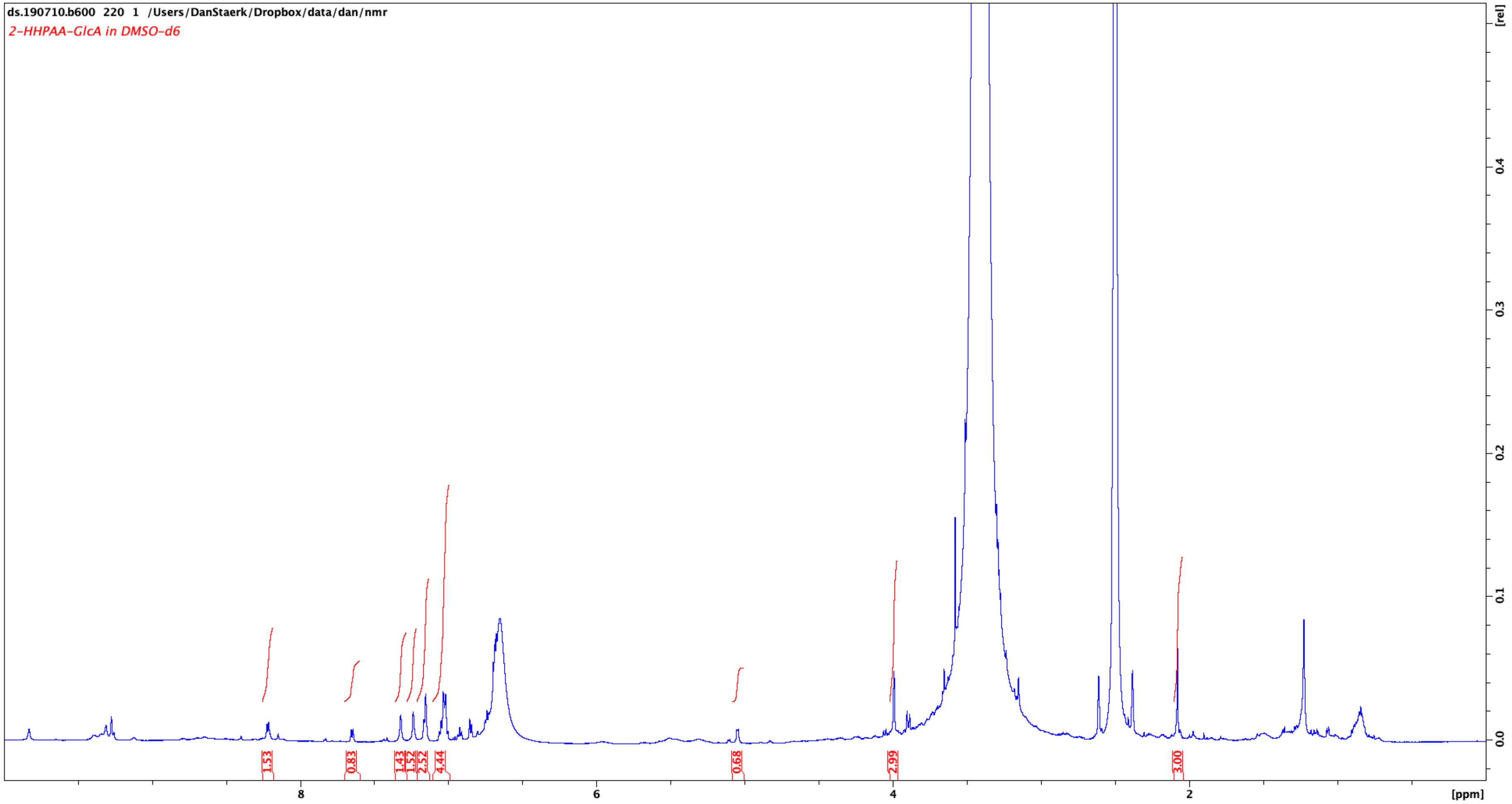

Spectrum 11. HSQC spectrum of **2-HPAA-glcA** in DMSO-d_6 (600 MHz)

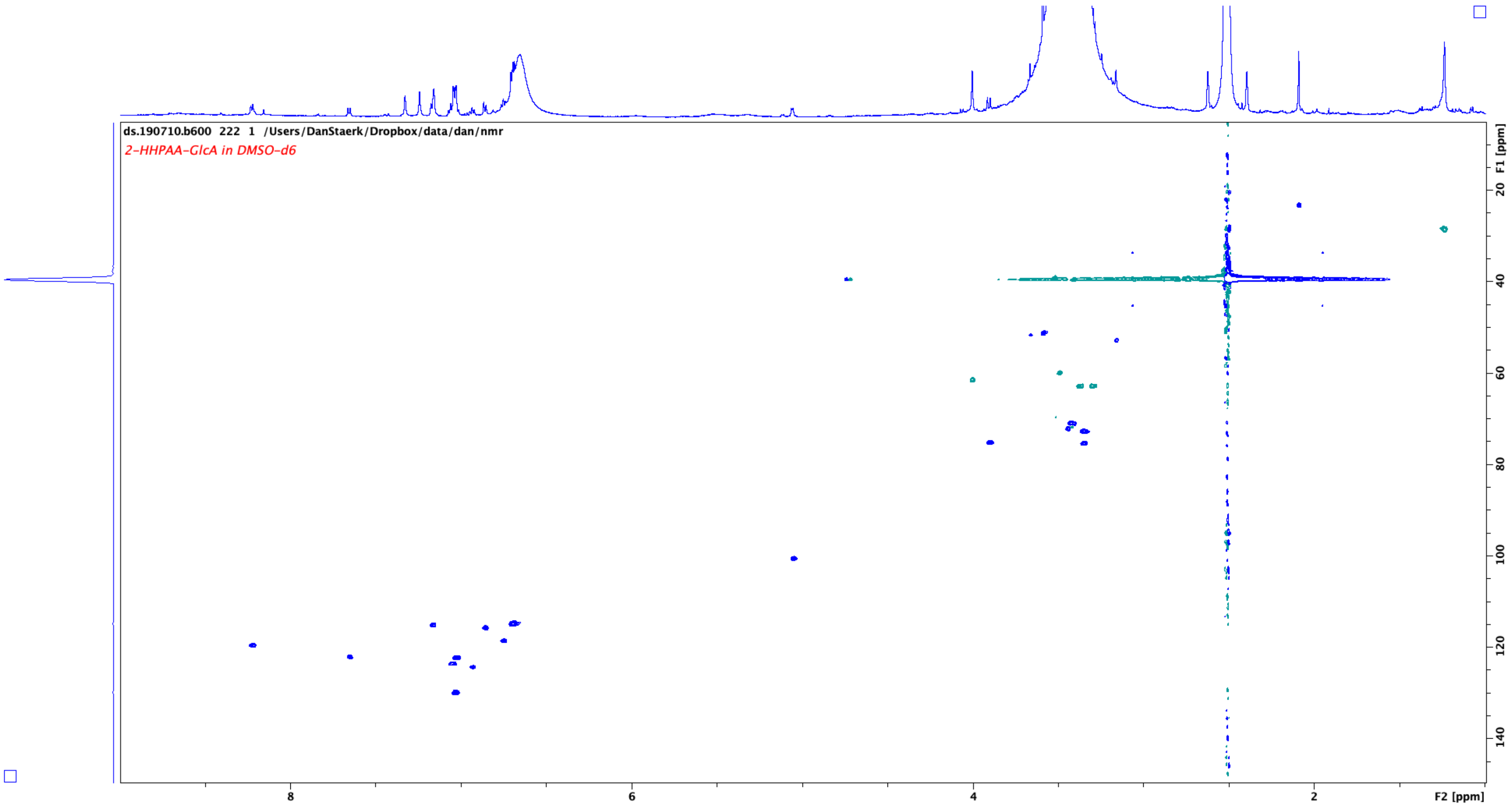

Spectrum 12. ROESY spectrum of **2-HHPAA-glcA** in DMSO-d_6 (600 MHz)

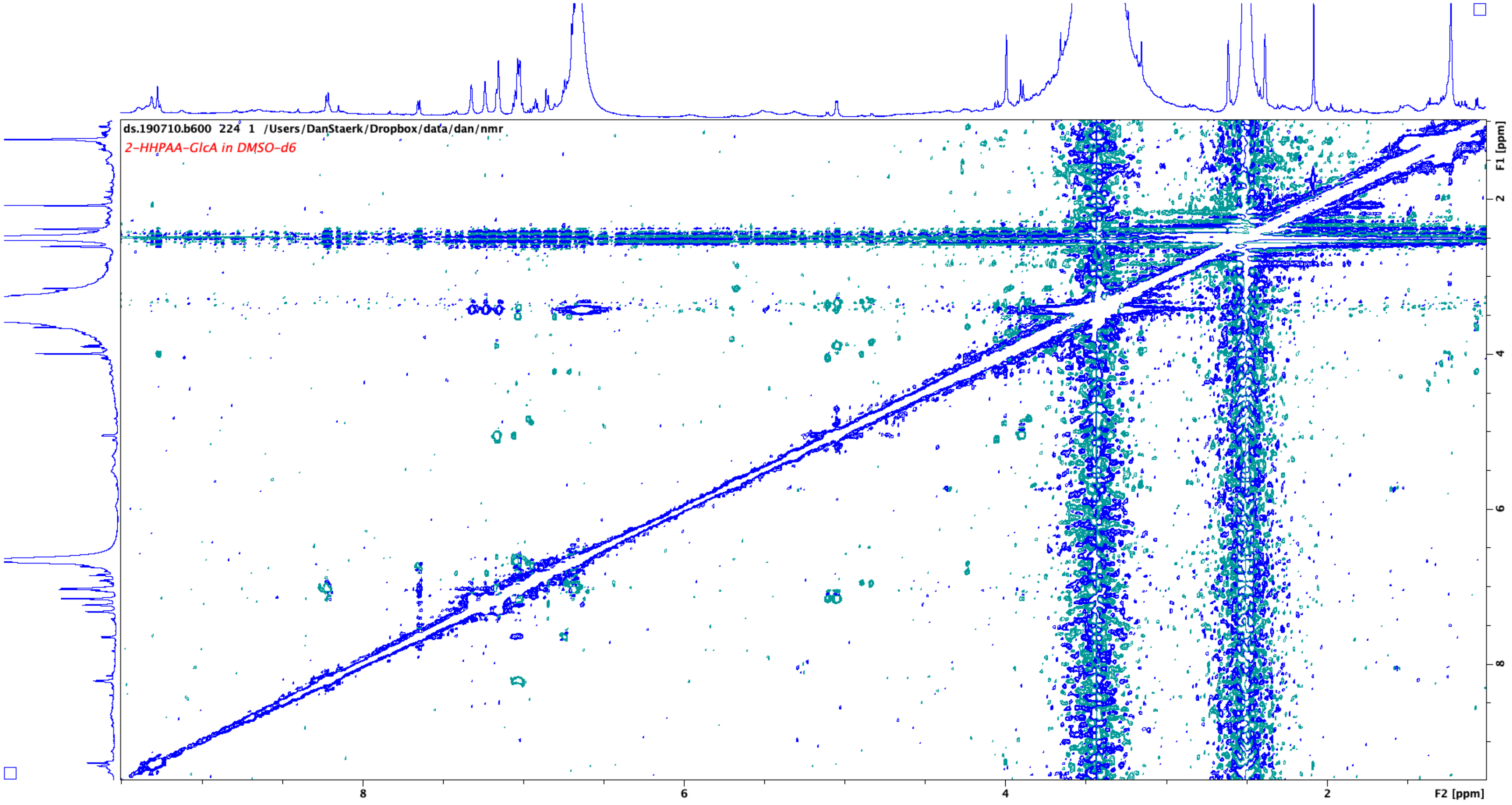

References for supplementary material

1. Bhattarai, B., Steffensen, S. K., Staerk, D., Laursen, B. B. & Fomsgaard, I. S. Data-dependent acquisition-mass spectrometry guided isolation of new benzoxazinoids from the roots of Acanthus mollis L. *International Journal of Mass Spectrometry* **474**, 116815 (2022).

2. EUROPEAN COMMISSION. *Guidance document on analytical quality control and method validation procedures for pesticide residues and analysis in food and feed. SANTE/11813/2017*. (2017).

3. European Medicines Agency. *ICH Topic Q 2 (R1) Validation of Analytical Procedures: Text and Methodology*. (1995).

4. Magnusson B and Örnemark U. *Eurachem Guide: The Fitness for Purpose of Analytical Methods – A Laboratory Guide to Method Validation and Related Topics*. (Eurachem, 2014).

5. Steffensen, S. K. *et al.* Benzoxazinoids in Prostate Cancer Patients after a Rye-Intensive Diet: Methods and Initial Results. *Journal of Agricultural and Food Chemistry* **64**, 8235–8245 (2016).
