# Supplementary figures and images for: An inverse association between plasma benzoxazinoid metabolites and PSA after rye intake in men with prostate cancer revealed with a new method
Source: Sci Rep. 2022 Mar 28;12:5260. doi: 10.1038/s41598-022-08856-z (PMC8960836; doi:10.1038/s41598-022-08856-z)

## Slide 1
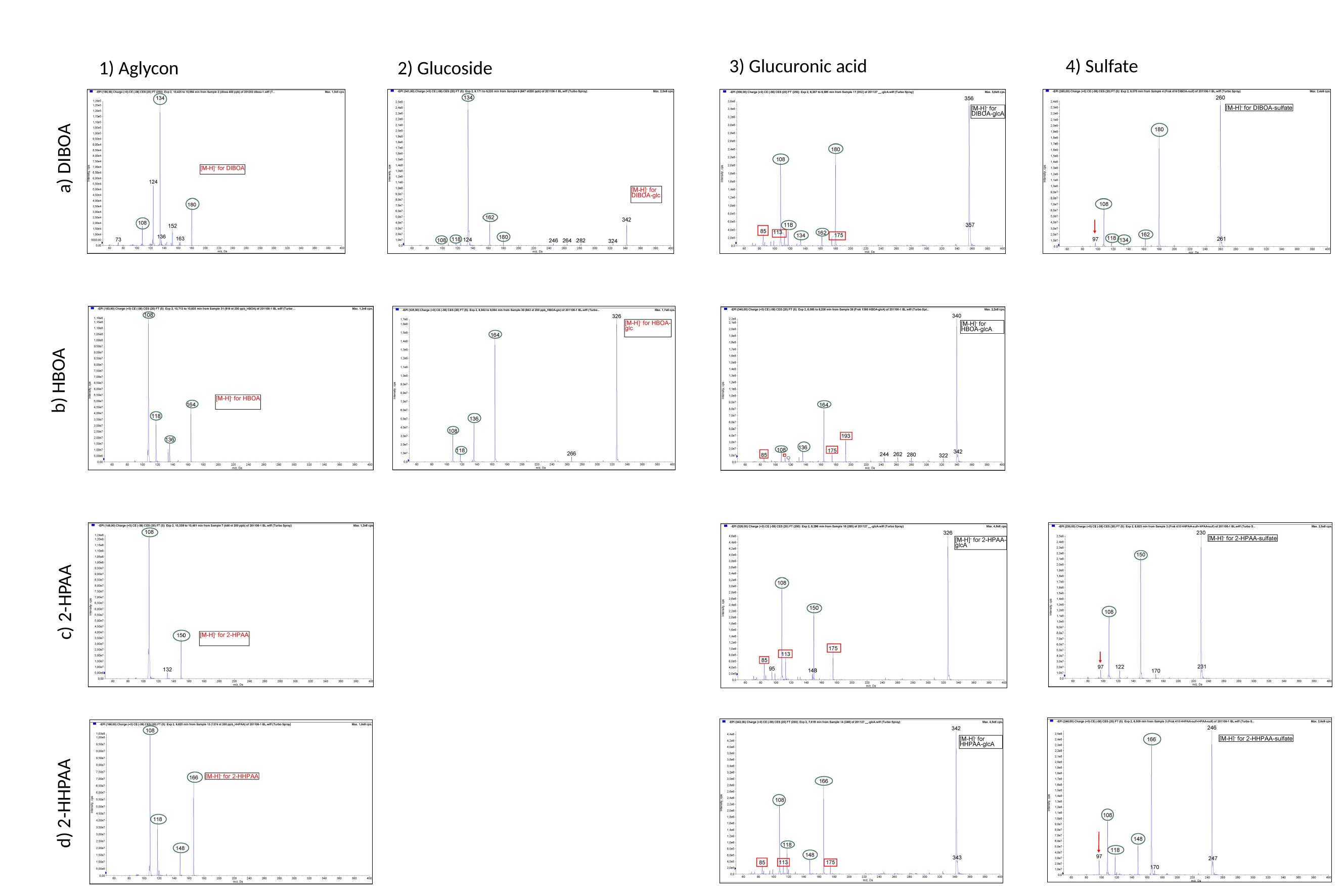

3) Glucuronic acid
4) Sulfate
1) Aglycon
2) Glucoside
a) DIBOA
b) HBOA
c) 2-HPAA
d) 2-HHPAA

Supplement: Supplementary file 2 — Supplementary Information 2. [file 41598_2022_8856_MOESM2_ESM.pptx]
